# Cuproptosis-related risk score predicts prognosis and characterizes the tumor microenvironment in colon adenocarcinoma

**DOI:** 10.3389/fonc.2023.1152681

**Published:** 2023-06-02

**Authors:** Jinyan Wang, Zhonghua Tao, Biyun Wang, Yizhao Xie, Ye Wang, Bin Li, Jianing Cao, Xiaosu Qiao, Dongmei Qin, Shanliang Zhong, Xichun Hu

**Affiliations:** ^1^ Department of Breast and Urologic Medical Oncology, Shanghai Medical College, Fudan University Shanghai Cancer Center, Shanghai, China; ^2^ Department of Pathology, Nanjing Jiangning Hospital, The Affiliated Jiangning Hospital of Nanjing Medical University, Nanjing, China; ^3^ Center of Clinical Laboratory Science, The Affiliated Cancer Hospital of Nanjing Medical University & Jiangsu Cancer Hospital & Jiangsu Institute of Cancer Research, Nanjing, China

**Keywords:** cuproptosis-related genes (CRGs), tumor microenvironment (TME), molecular subtypes, prognosis model, colon adenocarcinoma

## Abstract

**Introduction:**

Cuproptosis is a novel copper-dependent regulatory cell death (RCD), which is closely related to the occurrence and development of multiple cancers. However, the potential role of cuproptosis-related genes (CRGs) in the tumor microenvironment (TME) of colon adenocarcinoma (COAD) remains unclear.

**Methods:**

Transcriptome, somatic mutation, somatic copy number alteration and the corresponding clinicopathological data of COAD were downloaded from The Cancer Genome Atlas (TCGA) and Gene Expression Omnibus database (GEO). Difference, survival and correlation analyses were conducted to evaluate the characteristics of CRGs in COAD patients. Consensus unsupervised clustering analysis of CRGs expression profile was used to classify patients into different cuproptosis molecular and gene subtypes. TME characteristics of different molecular subtypes were investigated by using Gene set variation analysis (GSVA) and single sample gene set enrichment analysis (ssGSEA). Next, CRG Risk scoring system was constructed by applying logistic least absolute shrinkage and selection operator (LASSO) cox regression analysis and multivariate cox analysis. Real-time quantitative polymerase chain reaction (RT-qPCR) and immunohistochemistry (IHC) were used to exam the expression of key Risk scoring genes.

**Results:**

Our study indicated that CRGs had relatively common genetic and transcriptional variations in COAD tissues. We identified three cuproptosis molecular subtypes and three gene subtypes based on CRGs expression profile and prognostic differentially expressed genes (DEGs) expression profile, and found that changes in multilayer CRGs were closely related to the clinical characteristics, overall survival (OS), different signaling pathways, and immune cell infiltration of TME. CRG Risk scoring system was constructed according to the expression of 7 key cuproptosis-related risk genes (GLS, NOX1, HOXC6, TNNT1, GLS, HOXC6 and PLA2G12B). RT-qPCR and IHC indicated that the expression of GLS, NOX1, HOXC6, TNNT1 and PLA2G12B were up-regulated in tumor tissues, compared with those in normal tissues, and all of GLS, HOXC6, NOX1 and PLA2G12B were closely related with patient survival. In addition, high CRG risk scores were significantly associated with high microsatellite instability (MSI-H), tumor mutation burden (TMB), cancer stem cell (CSC) indices, stromal and immune scores in TME, drug susceptibility, as well as patient survival. Finally, a highly accurate nomogram was constructed to promote the clinical application of the CRG Risk scoring system.

**Discussion:**

Our comprehensive analysis showed that CRGs were greatly associated with TME, clinicopathological characteristics, and prognosis of patient with COAD. These findings may promote our understanding of CRGs in COAD, providing new insights for physicians to predict prognosis and develop more precise and individualized therapy strategies.

## 1 Introduction

Colon adenocarcinoma (COAD) is presently considered as one of the most common malignancies and the leading cause for mortality worldwide, resulting in more than 500,000 deaths every year (1). Although surgery, adjuvant/neoadjuvant chemotherapy, targeted therapy and immunotherapy have achieved certain efficacy, some patients still have a poor prognosis due to high recurrence and mortality rate (2). In recent years, more and more studies have aimed to provide a more personalized and accurate assessment of patient prognosis through a comprehensive analysis of the genomic and clinicopathological characteristics of specific tumors, with a view to potentially improving patient prognosis (3). Nonetheless, present biomarkers or methods are far from satisfactory to accurately predict outcome of patients with COAD.

Copper (Cu) is known as the third most abundant trace element in human body (4). It is traditionally considered as a redox-active transition metal which participated in the process from cellular respiration to pigmentation, acting through cytochrome c oxidase and tyrosinase (5). However, in the last decade, metalloallostery, a new form of protein regulation and nutrient sensing, has appeared to extend the function of Cu beyond the catalytic proteins to dynamic signaling molecules, which are the basis of cell biology affecting pathophysiological processes (6). Blood concentrations of Cu were significantly increased in multiple cancers, such as thyroid cancer, lung cancer, breast cancer and pancreatic cancer (7–10). In addition, Cu concentration was elevated in tissues of large bowel and oesophageal cancer (11). However, the blood concentration of Cu was decreased in patients with endometrial cancer (12). As a result, researches started to pay attention to the specific underlying mechanisms of Cu dys-homeostasis in cancers. Increasing evidence indicated that Cu dys-homeostasis might induce cytotoxicity and affect proliferation, apoptosis, and metastasis of tumors, thus resulting in cancer progression, partly through regulating kinases activation, lipolysis, potassium channels, BRAF, NF-κB and TGF-β signaling pathways (13–18). Most importantly, Tsvetkov et al. (19) recently claimed that cuproptosis was a kind of copper-dependent death and different from all other known programmed cell death (PCD). In terms of mechanics, Cu directly bound to the fatty acylation component of the tricarboxylic acid (TCA) cycle, thus leading to the accumulation of fatty acylation proteins and the subsequent loss of iron-sulfur cluster proteins, which leaded to protein-toxic stress and ultimately to cell death. Additionally, a total of 10 cuproptosis-related genes (CRGs), including PDHB, MTF1, FDX1, DLAT, PDHA1, LIAS, LIPT1, DLD, GLS and CDKN2A, were identified in this study. Based on Tsvetkov et al.’s findings, a growing number of researches have begun to investigate the relationships between CRGs and typical cancers. For instance, Zhang, Z., et al. (20) demonstrated prognostic features associated with cuproptosis in patients with hepatocellular carcinoma (HCC). Wang, W., et al. (21), identified a cuproptosis-related prognostic signature (H19, CYTOR, IGFBP2, KLRC2, C5orf38 and CHI3L1) for patients with glioma.

Tumor microenvironment (TME), which contains different immune and stromal cells and their secreted factors, has been recognized to cultivate a chronic inflammatory, immunosuppressive, and pro-angiogenic intra-tumoral atmosphere and is closely associated with patient outcomes and treatment efficacy (22). Distinct cuproptosis-related signatures were also found to be significantly associated with TME of kidney renal clear cell carcinoma (KIRC) (23), triple-negative breast cancer (TNBC) (24) and lung adenocarcinoma (LUAD) (25). However, due to tumor and corresponding TME heterogeneity, CRGs characteristics vary across cancers. In addition, studies of CRGs in COAD are limited.

In our study, we aimed to comprehensively analyze the relationship between CRGs and TME in COAD and construct a CRGs Risk scoring system to accurately predict COAD patient survival. The development of the scoring system provided physicians with new insights to design more effective and individualized treatment strategies.

## 2 Materials and methods

### 2.1 Data

Transcriptome and the corresponding clinicopathological data of COAD were downloaded from The Cancer Genome Atlas (TCGA) (https://portal.gdc.cancer.gov/) and Gene Expression Omnibus database (GEO) (https://www.ncbi.nlm.nih.gov/geo/). In detail, the TCGA cohort included 480 COAD tissues and 41 normal tissues. The GEO cohort containing GSE17536, GSE29623 and GSE39582, included 827 COAD samples. The detailed clinicopathological data of these COAD patients was presented in **Table S1**. The TCGA and GEO cohorts were combined by using “Combat” algorithm in R to eliminate batch effects before conducting subsequent analyses. Principal component analysis (PCA) was applied to validate the effect of batch effect removal by using the R package ggplot2. In order to verified the accuracy of model, we also downloaded transcriptome and the corresponding clinicopathological data of GSE40967 from GEO database, which contained 585 COAD sampes.

Additionally, we downloaded somatic mutation data of 454 tumor samples and copy number variation (CNV) data of 506 tumor samples from TCGA.

### 2.2 Difference analyses, survival analyses and correlation analyses of CRGs

A total of 10 CRGs (PDHA1, PDHB, FDX1, DLD, DLAT, MTF1, LIAS, LIPT1, GLS and CDKN2A) were obtained from the previous well-known publication of Tsvetkov et al. (19). Difference analyses of CRGs were conducted between tumor and normal tissues. Wilcoxon test was used to for statistical analysis. Survival and survminer R packages were used for survival analysis, the same as our previous study (26). Kaplan-Meier plot and cox regression analyze were further applied to evaluate the relationships between CRGs expression and patient overall survival (OS). Schoenfeld residuals were used to check the proportional assumption of COX model. Spearman correlation analyses were conducted to explore the interactions among CRGs

### 2.3 Consensus clustering analysis of CRGs

ConsensusClusterPlus R package was applied for consensus unsupervised clustering analysis. Patients were grouped into distinct molecular subtypes according to the expression of CRGs, and distinct gene subtypes according to the expression of prognostic differentially expressed genes (DEGs), derived from different molecular subtypes. The criteria included that the samples size in each set was relatively consistent and the cumulative distribution function (CDF) curve increased gradually and smoothly. After consensus clustering analysis, the intra-set association became stronger, while the inter-set association became weaker.

### 2.4 Associations among molecular subtypes, clinicopathological features and prognosis

We applied Kaplan-Meier plot and log-rank test to evaluate the associations between different molecular subtypes and patient survival. Correlation analyses between molecular subtypes and clinicopathological features were carried out to learn the clinical values of distinct molecular subtypes by using Chi-square test. The clinicopathological features contained age, gender, grade and tumor node metastasis (TNM) stage.

### 2.5 Relationships between molecular subtypes and TME

We downloaded the hallmark gene sets, including C2.CP.KEGG (186 gene sets) and C5.GO.Gene Ontology (10561 gene sets), from the Molecular Signatures Database (MSigDB) (https://www.gsea-msigdb.org/gsea/msigdb). Gene set variation analysis (GSVA) with the above two gene sets was conducted to explore the TME characteristics of different molecular subtypes. The adjusted P-value< 0.05 was considered statistically different. Additionally, the proportion of tumor-infiltrating immune cells (TICs) in tumor samples was calculated by using the deconvolution algorithm, which was also known as CIBERSORT (27). The gene expression signature matrix of TICs was downloaded from CIBERSORT platform (https://cibersortx.stanford.edu/). P-value for the deconvolution of each sample was obtained by using Monte Carlo sampling algorithm in R. A CIBERSORT P-value< 0.05 was considered suitable for further analysis. Single sample gene set enrichment analysis (ssGSEA) was used to evaluate the infiltration of TICs in different molecular subtypes.

### 2.6 Acquisition of DEGs from distinct molecular subtypes

DEGs of distinct molecular subtypes were acquired by applying limma package in R. The fold change of 1.5 and the adjusted P-value< 0.05 were considered qualified for searching DEGs. Gene Ontology (GO) and Kyoto Encylopedia of Genes and Genomes (KEGG) enrichment analysis of DEGs were carried out by using org.Hs.eg.db, ClusterProfiler, enrichplot, and ggplot2 packages in R. The adjusted P-value< 0.05 was deemed statistically significant.

### 2.7 Establishment of CRG Risk scoring system

Firstly, cox regression analyses of DEGs, achieved from different molecular subtypes, were carried out to seek those associated with patients’ prognosis. Secondly, patients were separated into different gene subtypes *via* consensus clustering analysis of prognostic DEGs expression. Thirdly, patients were randomly divided into the training (n=603) and testing (n=603) sets at a ratio of 1:1. Lastly, CRG Risk scoring system was established in the training set and verified in the testing set, GSE29263, GSE17536, GSE39582 and the combined set. Logistic least absolute shrinkage and selection operator (LASSO) cox regression analysis was carried out by applying Glmnet R package to decrease the risk of over-fitting. Next, we analyzed and cross-validated the varied trajectory of each independent variable. Multivariate Cox analysis was carried out to screen prognostic DEGs in the training group. The Risk score was calculated as follows:


CRG Riskscore=Σ(Expi∗coefi)

In detail, Expi indicated key prognostic DEGs expression and coefi indicated the coefficient of Risk. Correlation analysis between CRG Risk score and distinct subtypes was also carried out. Survival analysis between high- and low-risk sets was conducted by Kaplan-Meier plot and log-rank test. Receiver operating characteristic (ROC) curves were utilized to learn the sensitivity and specificity of the scoring system. Similarly, all of the testing group, GSE29263, GSE17536, GSE39582 and the combined group were classified into high- and low-risk groups, respectively, and further analyzed by Kaplan-Meier survival curves and ROC curves.

### 2.8 Tissue samples acquisition, real-time quantitative polymerase chain reaction and immunohistochemistry

A total of 8 sets of COAD and paired normal tissues were harvested from COAD patients at Nanjing Jiangning Hospital. The study was permitted by the Ethics Committee of Nanjing Jiangning Hospital (2021-03-048-K01). Total RNA extraction and RT-qPCR were performed as our previous study (28). The primers used for RT-qPCR are shown in **Table S2**. Slides (4μm) of formalin-fixed paraffin-embedded tissue sections were incubated with GLS (1:200; Cell Signaling Technology), NOX1 (1:200; Proteintech), HOXC6 (1:50; Affinity Biosciences), TNNT1 antibody (1:100; Invitrogen). The expression level was scored semiquantitatively based on staining intensity and distribution using the immunoreactive score (IRS) as described (29) and as following: IRS = SI (staining intensity) x PP (percentage of positive cells). SI was determined as 0, negative; 1, weak; 2, moderate; and 3, strong. PP was defined as 0, negative; 1, 1-20% positive cells; 2, 21-50% positive cells; 3, 51-100% positive cells. Ten visual fields from different areas of each sample were selected randomly for the IRS evaluation and the average IRS was calculated as final value.

### 2.9 Relationships between TME and distinct Risk score groups

Difference analyses of CRGs expression levels were carried out between high- and low- Risk groups. Wilcoxon test was used for comparison. We further conducted correlation analyses not only between TICs and risk scores, but also TICs and key prognostic Risk genes. An ESTIMATE algorithm was used to analyze the ratio of immune/stromal components in TME. The Immune Score, Stromal Score and ESTIMATE Score presented the ratio of immune component, the stromal component and the sum of the both, respectively. Difference analyses of Immune/Stromal/ESTIMATE Score were conducted between high- and low- Risk score sets. Wilcoxon test was used for comparison.

### 2.10 Microsatellite instability cancer stem cell, tumor mutation burden and somatic mutations in different Risk score sets

Difference and correlation analyses of MSI, TMB and CSC in distinct CRG Risk score groups were conducted to study the underlying associations. Maftools package in R was applied for the comparison of mutation frequency in different Risk score sets.

### 2.11 Drug susceptibility analyses

In order to study effectiveness of drugs in different Risk groups, pRRophetic package in R was used to calculate the semi-inhibitory concentration (IC50) values of drugs.

### 2.12 Development of a nomogram

We applied Rms package in R to establish a nomogram, which combined clinicopathological characteristics, patient survival and CRG Risk score. In the nomogram, a variable matched a score and the scores for all variables were added together to get an overall score. Calibration maps of the nomogram were developed to evaluate the consistency between predicted 1, 3, and 5-year survival rates and actual outcomes. ROC curve was drawn to understand the sensitivity and specificity of the scoring system.

### 2.13 Statistical analyses

All statistical analyses were conducted by using R version 4.2.1. Statistical significance was set at P-value< 0.05.

## 3 Results

### 3.1 Identification of CRGs in COAD

**Figure 1:**
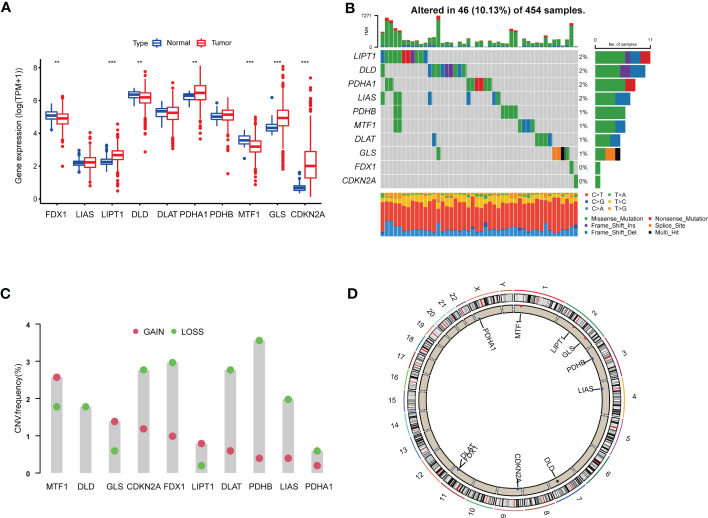
Genetic and transcriptional alterations of CRGs in colon adenocarcinoma. **(A)** The expression levels of 10 CRGs between 480 COAD samples and 41 normal samples. Wilcoxon test was used to compare two groups. **(B)** The maftool exhibited incidence of somatic mutations of CRGs in 454 COAD patients from TCGA database. **(C)** The CNV frequency of CRGs in 454 COAD samples from TCGA database. **(D)** Locations of CNV alterations on 23 chromosomes. P<0.05 was considered as significant importance. ** indicated P-value<0.01, *** indicated P-value<0.001.

We analyzed 10 CRGs in our study, including DLD, DLAT, PDHB, MTF1, PDHA1, FDX1, LIAS, LIPT1, GLS and CDKN2A. Difference analyses showed that 7 of 10 CRGs were dys-regulated in tumor samples compared with those in normal samples, among which LIPT1, PDHA1, GLS and CDKN2A were up-regulated, and FDX1, DLD and MTF1 were down-regulated (**Figure 1A**).

In order to further study the genetic and transcriptional alterations of CRGs in COAD, we generally analyzed the somatic mutation frequency of CRGs and found 10.13% mutation frequency in tumor samples (**Figure 1B**). LIPT1, DLD, PDHA1 and LIAS shared the highest mutation frequency (2%), followed by PDHB, MTF1, DLAT and GLS (1%). Both FDX1 and CDKN2A had no mutations in tumor tissues. We further examined CNV frequency in CRGs, among which DLD, CDKN2A, FDX1, DLAT, PDHB and LIAS had elevated copy number loss (**Figure 1C**). The detailed locations of these CRGs on chromosomes were shown in **Figure 1D**. As a result, we noted that CRGs had relatively common genetic and transcriptional variations in COAD tissues, which might affect oncogenesis.

### 3.2 Identification of cuproptosis-related molecular subtypes

**Figure 2:**
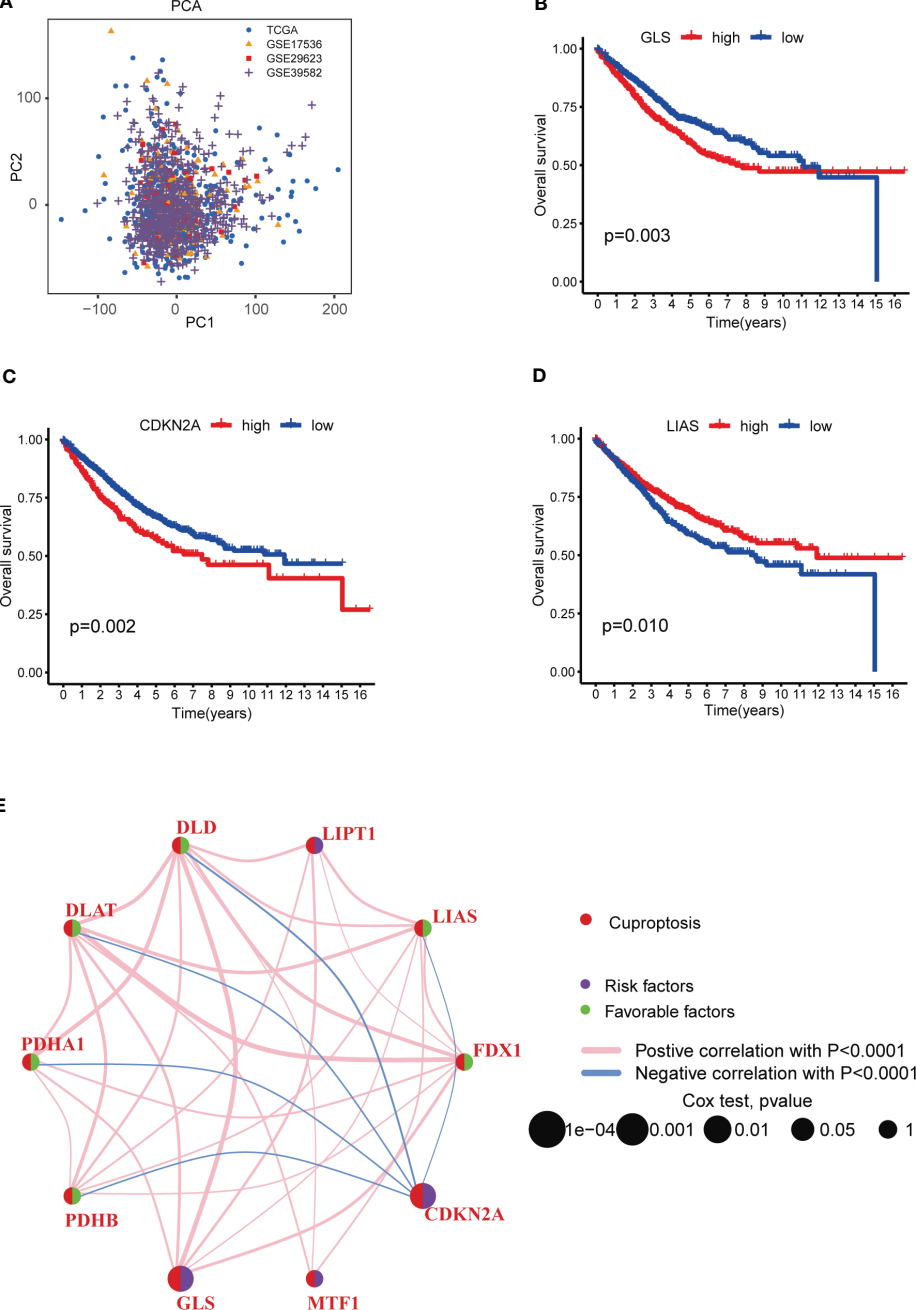
Survival analyses of CRGs and a comprehensive landscape of cuproptosis network in COAD patients from TCGA and GEO database. **(A)** PCA of TCGA, GSE17536, GSE29623 and GSE39582 after batch effect removal. **(B–D)** Survival analyses of CRGs (GLS, CDKN2A and LIAS) in COAD patients. Kaplan-Meier plot and log-rank tests were performed for survival analyses. Schoenfeld residuals was used to check the proportional assumption of COX model. **(E)** Mutual associations among CRGs in COAD samples. Spearman correlation analyses were used. The line between two CRGs indicated their interaction, and the stronger the correlation, the thicker the line. Pink line indicated positive correlation and blue line indicated negative correlation. P-value< 0.05 was considered to be statistically significant.

To learn the role of CRGs in oncogenesis of COAD, we combined expression patterns of CRGs and clinicopathological information of TCGA-COAD, GSE17536, GSE29623 and GSE39582 by using “Combat” algorithm to eliminate batch effects. PCA indicated that batch differences were well eliminated (**Figure 2A**). Kaplan-Meier plot revealed 3 of 10 CRGs were closely associated with patients’ OS, among which GLS and CDKN2A were negatively related, while LIAS was positively related (**Figures 2B–D**). Multivariate Cox regression analyses of CRGs also indicated that both GLS and CDKN2A were closely related with the survival of COAD patients (**Table 1**). Cuproptosis network generally described the complex interrelations among CRGs and the prognosis of patients with COAD (**Figure 2E**; **Table S3**).

**Table 1:** Multivariate Cox regression analyses of CRGs in COAD patients.

id	HR	HR.95L	HR.95H	P-value	km
CDKN2A	1.198237	1.0668866	1.3457599	0.002266	0.002461
GLS	1.302257	1.0853925	1.562451	0.004488	0.002666
LIAS	0.856896	0.718415	1.0220694	0.085936	0.009963
PDHB	0.792502	0.5976615	1.0508627	0.106232	0.002516
DLD	0.853752	0.6900535	1.0562847	0.145449	0.063517
PDHA1	0.887978	0.7283648	1.0825689	0.239915	0.066255
FDX1	0.884588	0.683168	1.1453929	0.352246	0.063822
DLAT	0.960967	0.7880859	1.1717723	0.693977	0.048397
LIPT1	1.02602	0.8300225	1.2682982	0.812277	0.112617
MTF1	1.027758	0.7957044	1.3274861	0.833903	0.10872

**Figure 3:**
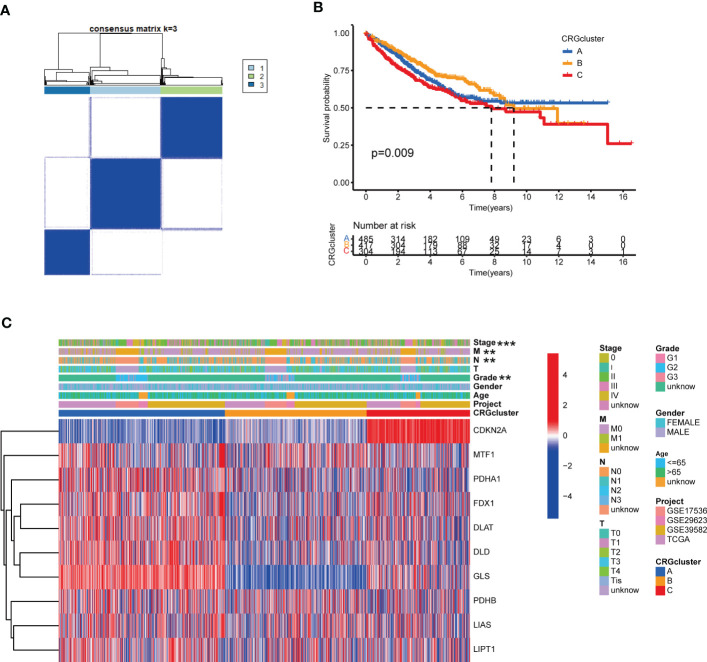
CRG molecular subtypes and their clinicopathological characteristics. **(A)** Identification of three molecular subtypes (k = 3) and their correlation area through consensus clustering analysis in COAD samples. **(B)** Survival analysis showed a significant difference in different molecular subtypes. Kaplan-Meier plot and log-rank tests were conducted for survival analyses. **(C)** The heat-map displayed the CRGs expression profile in distinct molecular subtypes, and the associations between clinicopathologic features and molecular subtypes. Chi-square test was used for the comparison. Red color indicated increased expression level and blue color indicated decreased expression level. P-value< 0.05 was considered to be statistically significant. ** indicated P-value<0.01, *** indicated P-value<0.001.

Considering the pervasive interrelations among CRGs, we used consensus clustering algorithm to divide patients into three groups based on the expression profile of CRGs. K=3 appeared to be an optimal choice for grouping samples into 3 sets, including molecular subtype A (n=511), B (n=444) and C (n=328) (**Figures 3A**, **S1A–I**, **Tables S4, 5**). Survival analysis revealed that patients in subtype C had the worst prognosis than those in subtype A or B (**Figure 3B**). The heat-map exhibited the expression profile of 10 CRGs in distinct molecular subtypes (**Figure 3C**). CDKN2A was obviously up-regulated in molecular subtype C, while PDHA1, FDX1, DLAT, DLD and GLS were greatly elevated in subtype A (**Figure 3C**). In addition, grade, N, M and stage were found to be significantly associated with cuproptosis molecular subtypes (**Figure 3C**).

### 3.3 Functional characteristics of TME in distinct molecular subtypes

**Figure 4:**
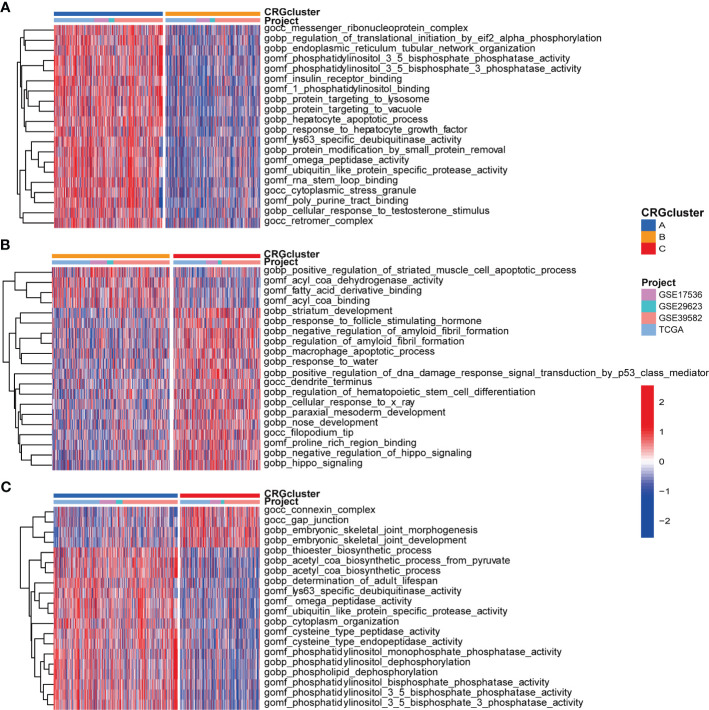
GO GSVA enrichment analyses indifferent molecular subtypes. **(A)** GO GSVA enrichment analyses between molecular subtype A and B **(B)** GO GSVA enrichment analyses between molecular subtype B and C **(C)** GO GSVA enrichment analyses between molecular subtype A and **(C)** Red color indicated more enriched in pathways and blue color indicated less enriched in pathways. Adjusted P-value<0.05 was considered to be statistically significant.

**Figure 5:**
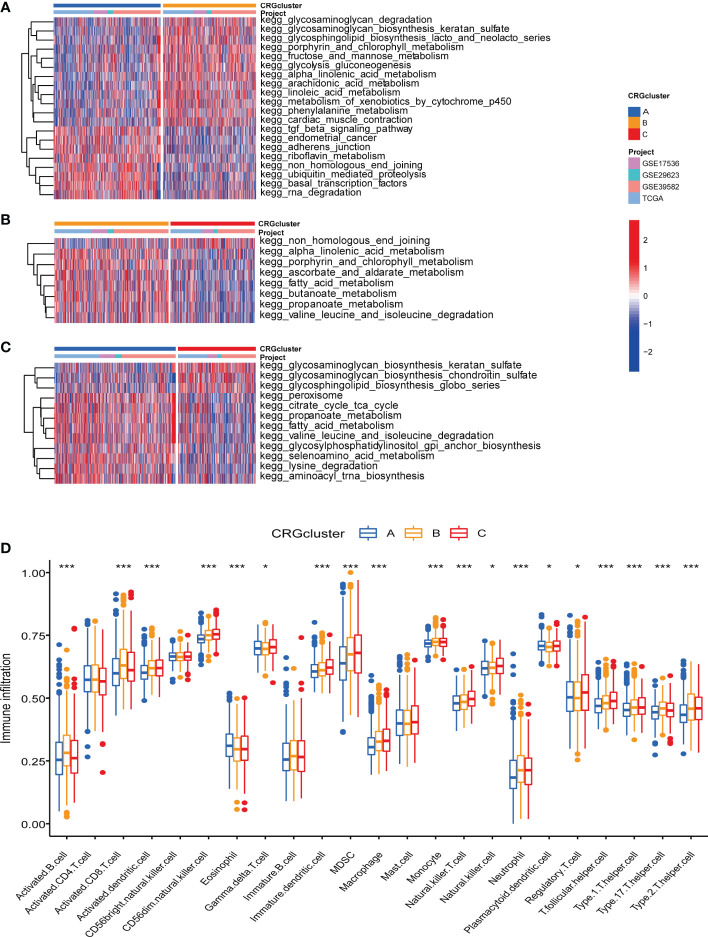
KEGG GSVA enrichment analyses and immune infiltration in different molecular subtypes. **(A)** KEGG GSVA enrichment analyses between molecular subtype A and B **(B)** KEGG GSVA enrichment analyses between molecular subtype B and C **(C)** KEGG GSVA enrichment analyses between molecular subtype A and C Red color indicated more enriched in pathways and blue color indicated less enriched in pathways. Adjusted P-value<0.05 was considered to be statistically significant. **(D)** ssGSEA indicated differences between the infiltration levels of TICs and distinct molecular subtypes. Pvalue<0.05 was considered to be statistically significant. * indicated P-value<0.05, *** indicated P-value<0.001.

We further performed GSVA enrichment analyses to explore the features of TME in different cuproptosis subtypes. GO GSVA enrichment analysis revealed that molecular subtype A was primarily enriched in messenger ribonucleoprotein complex, regulation of translational initiation by eif2 alpha phosphorylation and phosphatase activity, compared with subtype B (**Figure 4A**; **Table S6**). Subtype B was enriched in acyl coa binding, fatty acid derivative binding and acylcoa dehydrogenase activity, compared with subtype C (**Figure 4B**; **Table S6**). Subtype C was significantly enriched in embryonic skeletal joint morphogenesis, gap junction and connexin complex, compared with subtype A (**Figure 4C**; **Table S6**). Several biological pathways, such as endoplasmic reticulum tubular network organization, cellular response to zinc ion and mrna methylation were recurrent in the comparisons of subtype A and B, A and C, and B and C (**Table S7**). KEGG GSVA enrichment analysis indicated subtype A mainly participated in TGF-β signaling pathway, riboflavin metabolism and RNA degradation, compared with subtype B (**Figure 5A**; **Table S8**). Subtype B was primarily enriched in metabolic related pathways, including fatty acid metabolism, butanoate metabolism, porphyrin and chlorophyll metabolism, compared with subtype C (**Figure 5B**; **Table S8**). Subtype C was mainly enriched in glycosphingolipid biosynthesis globo series, glycosaminoglycan biosynthesis chondroitin sulfate and glycosaminoglycan biosynthesis keratan sulfate, compared with subtype A (**Figure 5C**; **Table S8**).

Regarding the complex functions of different molecular subtypes in TME, we next conducted ssGSEA between TICs and different subtypes to further identify tumor immune microenvironment (TIME) characteristics of COAD. The ratio of 23 TICs in each tumor sample was presented in **Table S9**. The result of ssGSEA suggested great difference between the infiltration of 19 TICs and distinct subtypes. In detail, the infiltration levels of eosinophil and plasmacytoid dendritic cell were elevated in subtype A, activated B cell, activated CD8 T cell, activated dendritic cell, monocyte and neutrophil were up-regulated in subtype B, and another 12 TICs were obviously raised in subtype C (**Figure 5D**).

According to above analyses, we primarily speculated that different subtypes took a different part in TME, especially TIME of COAD.

### 3.4 Identification of cuproptosis-related gene subtypes

**Figure 6:**
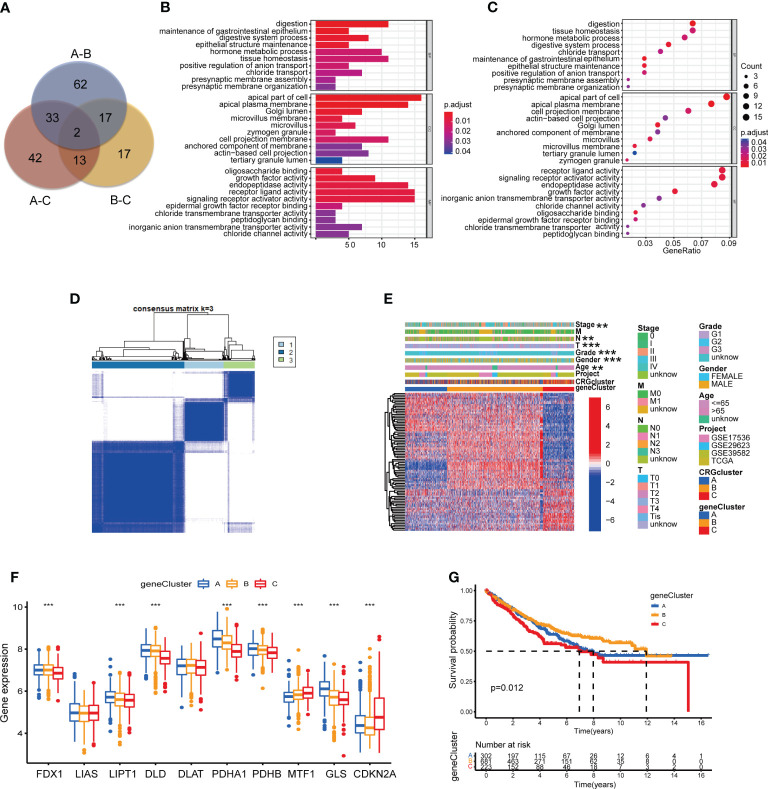
Identification of CRG gene subtypes based on 186 DEGs derived from different molecular subtypes. **(A)** The intersection of DEGs from the comparison between molecular subtype A and B, B and C, A and C **(B, C)** GO enrichment analyses of 186 DEGs from distinct molecular subtypes. Adjusted P-value<0.05 was considered to be statistically significant. **(D)** Identification of three gene subtypes (k = 3) and their correlation area through consensus clustering analysis according to the expression of 86 prognosis-related DEGs. **(E)** The heat-map presented the gene profiles in distinct gene subtypes, and the correlations between clinicopathologic characteristics and distinct gene subtypes. Chi-square test was used for the comparison. P-value< 0.05 was considered to be statistically significant. **(F)** Difference analyses of CRGs expression in different gene subtypes. Pvalue< 0.05 was considered to be statistically significant. **(G)** Survival analysis of three gene subtypes. Kaplan-Meier plot and log-rank tests were conducted for survival analyses. P-value< 0.05 was considered to be statistically significant. ** indicated P-value<0.01, *** indicated P-value<0.001.

As the potential role of different molecular subtypes in TME of COAD, we further explore the underlying biological behavior of different subtypes through seeking for DEGs. We identified 114 DEGs derived from subtype A and B, 90 DEGs from subtype A and C, 49 DEGs from subtype B and C (**Table S10**). Finally, a total of 186 DEGs were obtained for further analyses through combination (**Figure 6A**; **Table S11**). GO enrichment analysis demonstrated that 186 DEGs mainly participated in signaling pathways associated with digestion, such as maintenance of gastrointestinal epithelium and digestive system process (**Figures 6B, C**; **Table S12**). Univariate Cox regression analysis was performed to seek DEGs of prognostic value and finally identified 86 DEGs associated with patients’ OS, which were analyzed in the following section (**Table S13**). According to 86 prognostic DEGs expression, consensus clustering analysis was carried out to separate patients into 3 sets, namely gene subtype A (n=310), B (n=729) and C (n=244) (**Figures 6D**, **S2A–I**; **Tables S14, 15**). Distinct gene subtypes showed great differences in the expression levels of both prognostic DEGs and 8 CRGs (FDX1, LIPT1, DLD, PDHA1, PDHB, MTF1, GLS and CDKN2A) (**Figures 6E, F**; **Tables S16, 17**). In addition, cuproptosis gene subtypes were closely related with age, gender, grade, and T and N stage of COAD patients (**Figure 6E**). Survival analysis revealed that patients of gene subtype B had a better prognosis, compared with those of subtype A or C (**Figure 6G**).

### 3.5 Construction and validation of CRG Risk scoring system

**Figure 7:**
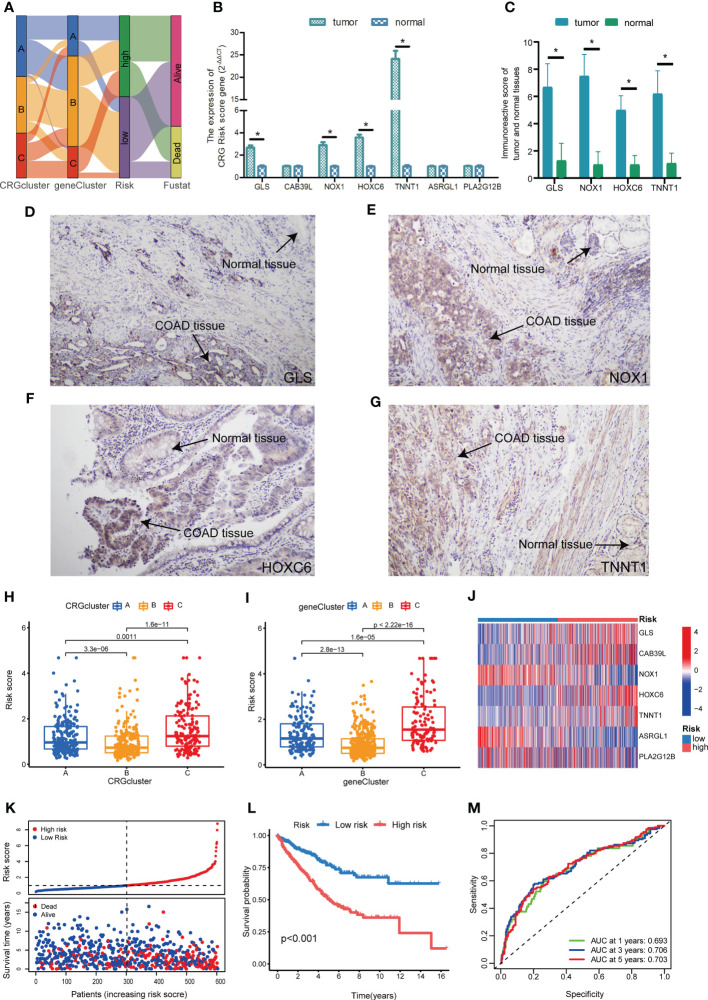
Construction of CRG Risk scoring system in the training group. **(A)** Alluvial diagram of patients’ distributions in groups with different molecular subtypes, gene subtypes, Risk scores and survival outcomes. **(B)** The expression of 7 key genes between COAD and paired normal tissues. **(C)** Immunoreactive score of key genes between tumor and normal tissues. **(D)** The expression of GLS in COAD tissues and normal tissues. **(E)** The expression of NOX1 in COAD tissues and normal tissues. **(F)** The expression of HOXC6 in COAD tissues and normal tissues. **(G)** The expression of TNNT1 in COAD tissues and normal tissues. **(H)** Difference analysis of CRG Risk score in different molecular subtypes. **(I)** Difference analysis of CRG Risk score in different gene subtypes. **(J)** Heat-map displayed five scoring genes expression profile in different risk sets of the training group. **(K)** Ranked dot and scatter plot of CRG Risk score distribution and patient survival in the training group. **(L)** Survival analysis between high- and low-Risk score groups in the training set. Kaplan-Meier plot and log-rank tests were conducted for survival analyses. **(M)** ROC curve predicted the sensitivity and specificity of 1-, 3-, and 5-year survival according to CRG Risk score in the training group. P-value< 0.05 was considered to be statistically significant. * indicated P-value<0.05.

To study the prognostic value of CRGs in COAD, we further constructed CRG Risk scoring system based on different molecular and gene subtypes. First, we applied “caret” package in R to randomly separate COAD patients into the training (n=603) and testing (n=603) groups at a ratio of 1:1. The clinicopathological characteristics of patients in the training and testing group were consistent (**Table S18**). Second, LASSO and multivariate Cox analyses were conducted to identify optimum prognostic signature based on 86 DEGs expression (**Figure S3**). Finally, CRG Risk scoring system was established through multivariate Cox regression analysis in the training set, the formula was as follow: Risk score = (0.30346935571892* expression of GLS) + (0.285346929484159 * expression of CAB39L) + (-0.171967289741126* expression of NOX1) + (0.149406405352724 * expression of HOXC6) + (0.128828618079011 * expression of TNNT1) + (-0.305462961248901* expression of ASRGL1) + (-0.142788274274145* expression of PLA2G12B). We classified patients into two groups, namely high- and low-Risk score sets, according to the calculation of Risk score in each tumor sample. **Figure 7A** presented the specific classifications of patients in the training set, including three cuproptosis molecular subtypes, three gene subtypes and two CRG Risk score sets. The detailed information of 7 key cuproptosis-related risk genes, Risk score and survival features in training and testing groups was displayed in **Tables S19, 20**. The results of difference analyses indicated that all of the expression of GLS, NOX1, HOXC6, TNNT1 and PLA2G12B were increased in tumor tissues, compared with those in normal tissues (**Figure S4**). Among these five genes, GLS and HOXC6 were negatively associated with patients’ survival, while NOX1 and PLA2G12B were positively related. RT-qPCR and IHC indicated the same result (**Figures 7B–G**). Difference analyses in the training set showed Risk score was extremely increased in both molecular subtype C and gene subtype C and decreased in both molecular subtype B and gene subtype B (**Figures 7H, I**). The heat-map presented a great difference of 7 key Risk score gene expression profile between high- and low-Risk score sets in the training group (**Figure 7J**). The scattergram of patients’ survival in different Risk score groups revealed that COAD patients’ survival got worse, while Risk score increased (**Figure 7K**), which was also proven by Kaplan-Meier survival curves (**Figure 7L**). In addition, area under the time-concentration curve (AUC) values of 1-, 3-, and 5-year survival rates of CRG Risk score in the training set were 0.693, 0.706, and 0.703, respectively, signifying both relative high sensitivity and specificity (**Figure 6M**).

To verify the accuracy of the scoring system, we further calculated Risk score according to the above Risk score formula, in the testing group, individual GSE17536, GSE29623, GSE39582, GSE40967, respectively (**Tables S21–24**). Patients were respectively divided into distinct cuproptosis molecular subtypes, gene subtypes and Risk score sets, the same as which in the training set (**Figures S5–8A**). Risk score showed a great difference in both molecular subtypes and gene subtypes of the testing group, individual GSE17536, GSE29623, GSE39582 (**Figures S5–8B, C**). The expressions of 7 key Risk scoring genes in different Risk group were shown in **Figures S5–8 D**; **9A**, respectively. Both scattergram and Kaplan-Meier survival curves showed that high Risk score predicted poor survival in testing group, individual GSE17536, GSE39582 and GSE40967 (**Figures S5E, F, S7–8E, F, S9B, C**). However, in GSE29623, survival analysis revealed that Risk score was not associated with patients’ survival, which might be related with the small sample size (**Figure S6F**). We further plot ROC curves to confirm the sensitivity and specificity of the scoring system and found relatively high AUC values in the cohorts of validation, indicating the system as an accurate predictor for patients’ survival (**Figures S5–8G, 9D**).

### 3.6 Associations between TME and the CRG Risk score

**Figure 8:**
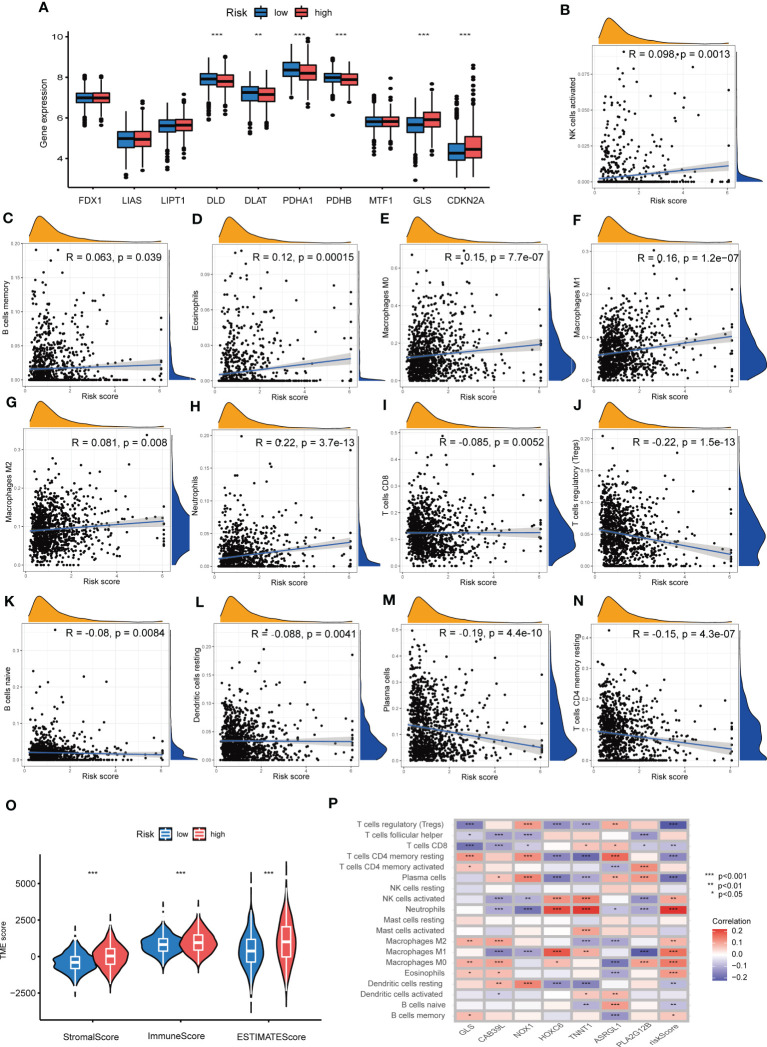
Associations between TME and CRG Risk score. **(A)** Difference analyses of CRGs expression in the high- and low-Risk score groups. **(B–N)** Correlation analyses between CRG Risk score and TICs. **(O)** Difference analyses between CRG Risk score and immune/stromal/estimate scores. **(P)** Correlation analyses between the abundance of TICs and seven key Risk scoring genes in the proposed model. P-value< 0.05 was considered to be statistically significant.

Difference analyses of CRGs indicated that 6 CRGs showed a great difference in distinct Risk score sets. To be specific, GLS and CDKN2A expression were increased, while DLD, DLAT, PDHA1 and PDHB expression were decreased in high-Risk score group, compared with those in low-Risk group (**Figure 8A**). In order to learn the relationships between CRG Risk score and TICs in TME of COAD, correlation analyses were carried out and suggested that CRG Risk score was positively associated with activated NK cells, memory B cells, eosinophils, M0 macrophages, M1 macrophages, M2 macrophages, and neutrophils, while negatively associated with CD8 T cells, regulatory T cells (Tregs), naïve B cells, resting dendritic cells, plasma cells and CD4 memory resting T cells (**Figures 8B–N**). Furthermore, all of immune, stromal and estimate score were higher in high-Risk score set than those in low-Risk score set (**Figure 8O**). Most immune cells were greatly associated with seven prognostic genes (**Figure 8P**). Consequently, CRG Risk score might be associated with TME of COAD.

### 3.7 Associations among MSI, CSC, TMB, somatic mutations and CRG Risk score

**Figure 9:**
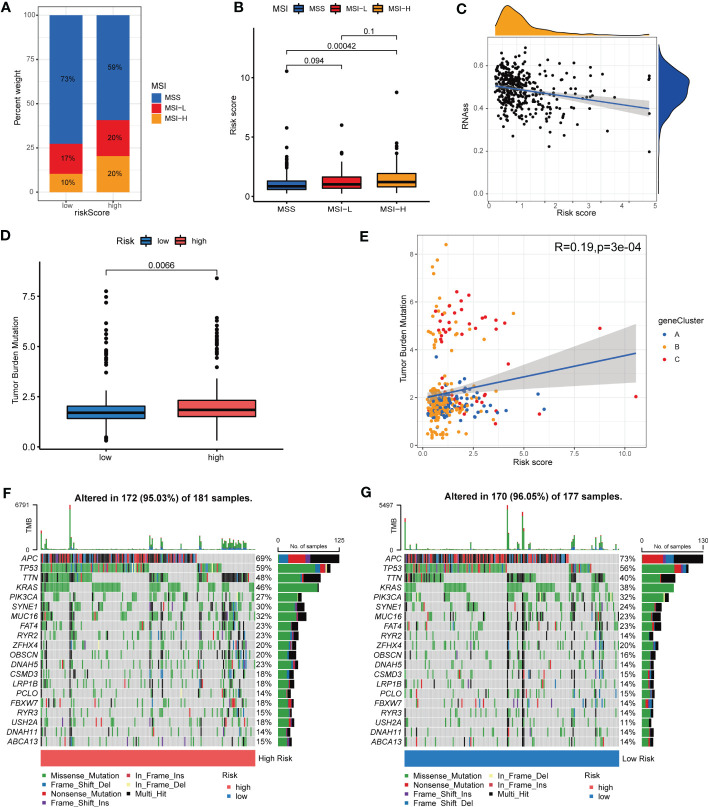
Associations among MSI, TMB, CSC and CRG Risk score. **(A)** The distribution of MSI in different Risk score groups. **(B)** Difference analysis between CRG Risk score and MSI. **(C)** Correlation analysis between CRG Risk score and CSC index. **(D)** Difference analysis of TMB in distinct CRG Risk score groups. **(E)** Correlation analysis between CRG Risk score and TMB. **(F-G)** The waterfall plot of somatic mutation characteristics in high- and low-Risk score groups. P-value< 0.05 was considered to be statistically significant.

Up to data, limited molecular markers are available to lead therapeutic decisions for patients with COAD, among which MSI, CSC, TMB and somatic mutations appeared to be the most promising. An increasing number of research revealed that patients with high microsatellite instability (MSI-H) tumor might benefit from immune checkpoint inhibitors (ICIs) in COAD (30, 31). As a result, we assessed the MSI status and found that in the low-risk group, 73% were MSS, 17% were low microsatellite instability (MSI-L), and 10% were MSI-H, while in the high-risk group, 59% were MSS, 20% were MSI-L, and 20% were MSI-H (**Figure 9A**). The results indicated that patients with high-risk shared a higher MSI-H frequency. **Figure 9B** suggested that patients bearing MSI-H tumors appeared to have a higher Risk score, compared with those with MSS. This might be related with better treatment outcomes of ICIs. Additionally, crosstalk between immune cells and CSCs, another important indicator of TIME, takes a great part in tumor progression (32). As presented in **Figure 9C**, CRG Risk score was negatively associated with CSC index, indicating COAD cells with high CRG Risk scores had less difference in stem cell properties and higher cell differentiation than those with low-risk scores. TMB, as an indicator of the number of tumor mutations, is known to be closely associated with patients’ immunotherapy benefits (33). Differential analysis indicated that TMB in high-risk group was significantly higher than that in low-risk group (**Figure 9D**). Correlation analysis also suggested that TMB was positively associated CRG Risk score (**Figure 9E**). Maftools of somatic mutations showed that the top 10 mutant genes in the high-risk and low-risk groups were APC, TP53, TTN, KRAS, PIK3CA, SYNE1, MUC16, FAT4, RYR2 and ZFHX4, respectively (**Figures 9F, G**).

### 3.8 Drugs susceptibility analysis in distinct Risk score groups

To investigate the predictive value of CRG Risk score in drug sensitivity, we used pRRophetic R package to calculate the IC50 values of various drugs (**Figures S10, 11**; **Table 2**). Both drugs under clinical use and clinical trials were included in our analyses. Various drugs were divided into different groups, such as AKT inhibitor, AMPK activator, Bcr-Abl inhibitor, BTK inhibitor, EGFR inhibitor, MAPK inhibitor, mTOR inhibitor, TrkA inhibitor, Topoisomerase inhibitor, Microtubule assosiated inhibitor, XIAP inhibitor, TNF inhibitor and so on. In particular, patients of low-Risk score set showed increased IC50 value for AMPK activator (AICAR), Bcl-2 inhibitor (TW.37, Obatoclax.Mesylate and ABT.263), BRAF inhibitor (PLX4720), c-Kit inhibitor (AMG.706), DNA Synthesis inhibitor (Cytarabine, Bleomycin and Gemcitabine), HSP90 inhibitor (AUY922), ITK inhibitor (BMS.509744), MEK inhibitor (CI.1040 and RDEA119), PARP inhibitor (AG.014699 and AZD.2281) and ROCK inhibitor (GSK269962A). In addition, patients of high-Risk score set showed increased IC50 value for AKT inhibitor (AKT.inhibitor.VIII and A.443654), CDK inhibitor (Roscovitine), Raf/VEGFR/c-Kit inhibitor (Sorafenib), Her-2 inhibitor (Lapatinib) and EGFR inhibitor (Erlotinib and BIBW2992).

**Table 2:** Drug susceptibility in patients of the low- and high-score groups.

Drugs		Low-score group	High-score group
AKT inhibitor	AKT.inhibitor.VIII		+
	A.443654		+
AMPK activator	AICAR	+	
Aurora Kinase inhibitor	ZM.447439	+	
	VX.680		+
Bcl-2 inhibitor	TW.37	+	
	Obatoclax.Mesylate	+	
	ABT.263	+	
Bcr-Abl inhibitor	Nilotinib	+	
	AP.24534	+	
	Dasatinib	+	
	Imatinib	+	
BRAF inhibitor	PLX4720	+	
BTK inhibitor	LFM.A13	+	
CDK inhibitor	Roscovitine		+
CHK inhibitor	AZD7762	+	
c-Kit inhibitor	AMG.706	+	
Raf/VEGFR/c-Kit inhibitor	Sorafenib		+
DNA Synthesis inhibitor	Cytarabine	+	
	Bleomycin	+	
	Gemcitabine	+	
DNA crosslinker/apoptosis inducer	Cisplatin	+	
EGFR inhibitor	Erlotinib		+
	BIBW2992		+
FAK inhibitor	PF.562271	+	
FGFR inhibitor	PD.173074	+	
FTase inhibitor	FTI.277	+	
Proteosome inhibitor	Z.LLNle.CHO	+	
GSK-3 inhibitor	CHIR.99021	+	
	SB.216763	+	
HDAC inhibitor	Vorinostat	+	
	MS.275		+
Hedgehog inhibitor	GDC.0449	+	
Her-2 inhibitor	Lapatinib		+
HSP90 inhibitor	AUY922	+	
ITK inhibitor	BMS.509744	+	
JNK inhibitor	JNK.Inhibitor.VIII	+	
	JNK.9L	+	
	AS601245	+	
MAPK inhibitor	VX.702	+	
MDM2 inhibitor	JNJ.26854165	+	
MEK inhibitor	CI.1040	+	
	RDEA119	+	
mTOR inhibitor	Temsirolimus	+	
	Rapamycin		+
	NVP.BEZ235	+	
	AZD8055	+	
PAK inhibitor	IPA.3	+	
PARP inhibitor	AG.014699	+	
	AZD.2281	+	
TBK1 and PDK1 inhibitor	BX.795	+	
PI3K inhibitor	AZD6482	+	
	GDC0941	+	
	NVP.BEZ235	+	
PKC inhibitor	Midostaurin	+	
PLK inhibitor	BI.2536		+
	GW843682X		+
PPAR inhibitor	FH535	+	
Rac inhibitor	EHT.1864		+
Raf inhibitor	AZ628	+	
ROCK inhibitor	GSK269962A	+	
RPTK inhibitor	CEP.701	+	
RSK inhibitor	BI.D1870	+	
	PF.4708671		+
	CMK	+	
RXR activator	Bexarotene	+	
Src inhibitor	A.770041	+	
	AZD.0530	+	
	Bosutinib	+	
Syk inhibitor	BAY.61.3606	+	
TNF inhibitor	Lenalidomide	+	
TrkA inhibitor	GW.441756		+
VEGFR inhibitor	Axitinib	+	
	Pazopanib	+	
PPM1D/Wip1 inhibitor	CCT007093	+	
XIAP inhibitor	Embelin	+	
Topoisomerase I inhibitor	Camptothecin	+	
Topoisomerase II inhibitor	Doxorubicin	+	
	Etoposide	+	
Microtubule Assosiated inhibitor	Docetaxel	+	
	Vinblastine	+	
Microtubule stabilizer	Paclitaxel		+
SER Ca2+-ATPase inhibitor	Thapsigargin	+	
	Metformin		+
Cuproptosis inducer	Elesclomol	+	
ARFGAP1 inhibitor	QS11	+	
Chloride Channel inhibitor	Shikonin	+	
eIF2α Dephosphorylation inhibitor	Salubrinal		+
SHP PTP inhibitor	NSC.87877	+	
DNA-PK inhibitor	NU.7441	+	

“+”: Indicated up-regulated sensitivity.

However, drugs that target the same site may have opposite effects in different risk groups. For example, patients with low CRG risk scores had increased IC50 values for Aurora kinase inhibitors (ZM.447439) and decreased IC50 values for aurora kinase inhibitors (VX.680). HDAC inhibitors (Vorinostat) had increased IC50 values and HDAC inhibitors (MS.275) had decreased IC50 values in the low-risk score set. mTOR inhibitors (Temsirolimus, NVP.BEZ235 and AZD8055) presented better drug sensitivity in low-risk score set, while mTOR inhibitor (rapamycin) had the opposite. RSK inhibitors (BI. D1870 and CMK) and PF.4708671. RSK inhibitor (BI.D1870 and CMK) and PF.4708671 also showed the opposite drug susceptibility between different risk score sets.

### 3.9 Construction of a nomogram for the prediction of COAD patient’s survival

**Figure 10:**
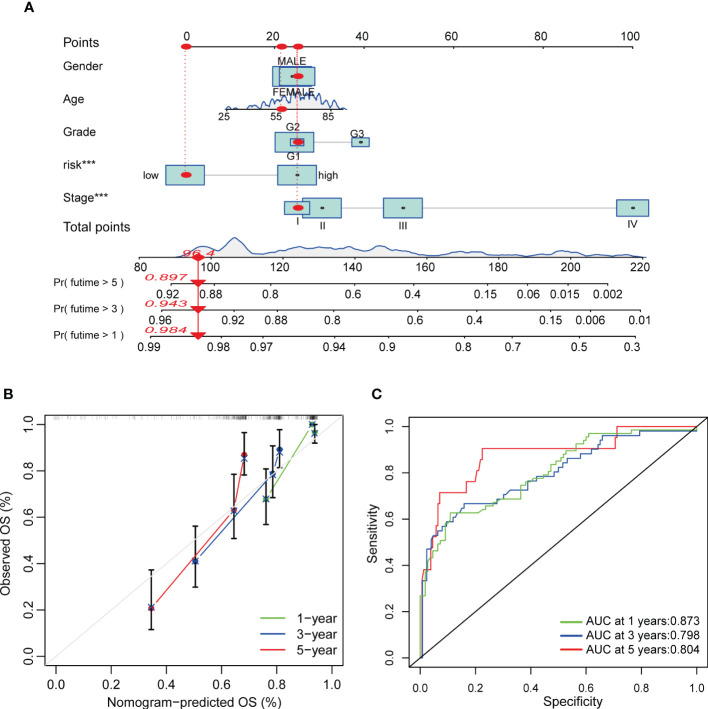
Construction and validation of a nomogram in COAD patients. **(A)** Nomogram for predicting the 1-, 3-, and 5-year OS of COAD patients. **(B)** Calibration curves of the nomogram. **(C)** ROC curves for predicting the 1-, 3-, and 5-year OS of COAD patients.

Regarding the important role of Risk score in patients’ survival, we constructed a nomogram combining CRG Risk scores and clinicopathological characteristics to predict 1, 3, and 5-year survival rates of COAD patients (**Figure 10A**). The calibration graph showed that the nomogram functioned well in predicting patients’ survival compared to an ideal model (**Figure 10B**). The AUC values of 1, 3, and 5-year survival rates of nomogram were 0.873, 0.798, and 0.804, respectively, suggesting both relatively high sensitivity and specificity (**Figure 10C**).

## 4 Discussion

COAD is a global health problem. Despite continuous improvement of early screening and treatment strategies, the survival of patients with advanced COAD remains poor (1). Previous research suggested genomic susceptibility contributed to the occurrence and development of COAD (34–37). For example, BRAF V600E and KRAS mutations were significantly related with poor prognosis of patients with microsatellite-stable COAD (38). However, risk factors affecting patients’ survival varied and the above risk factors predicting the prognosis of patients were not yet satisfactory.

TME is a highly complex ecosystem (39). The subtle interactions between tumor cells and co-existing immune cells in TME determine tumor’s natural history. Based on pioneer studies on TME, the two most widely applied ICIs, blocking cytotoxic-T-lymphocyte-associated protein 4 (CTLA-4) and targeting programmed cell death 1 (PD-1) or programmed cell death ligand 1 (PD-L1), emerged as exciting treatment strategies across various malignancies in the last decade (40). ICIs showed impressive anti-tumor efficacy in COAD patients bearing tumors with the expression of PD-L1, deficient mismatch repair (dMMR), MSI-H, or high TMB (41, 42). Whereas the number of COAD patients who benefit from ICIs is limited due to primary and acquired resistance. Therefore, comprehensive knowledge of changes in genomic, transcriptome and somatic mutations in TME is of great significance for the prevention, treatment and prognosis assessment of COAD.

PCD, also termed as RCD, is a form of cell death that can be regulated by multiple biomacromolecules, thus leading to biochemical and morphological alterations which are depend on energy (43). Increasing evidence has indicated that RCD is the key features of tumorigenesis, which may ultimately affect therapeutic strategies in cancers (44). RCD subroutines containing apoptosis, necroptosis, autophagy, pyroptosis, ferroptosis, lysosome-dependent cell death (LCD), alkaliptosis and NETosis have been identified and are being extensively investigated in a variety of malignancies (45). For instance, interactions between specific pyroptosis-related subtypes and TME greatly influenced patients’ prognosis (46). Dividing cancer patients into different subtypes according to their genomic features allows us to more accurately predict drug susceptibility and patient outcome, helping physicians design more precise and individualized treatment strategies (47–49).

Cu is an essential micronutrient participated in multiple fundamental biological processes (50). Aberrant Cu homeostasis (ACH) is associated with tumor growth, metastasis, and drug resistance due to its role in oxidative stress and chronic inflammation (51). A higher Cu level indicated a higher risk of colorectal cancer (52). In addition, Cu chelator exhibited great antitumor activity in various cancers, such as esophageal cancer, triple-negative breast cancer and COAD (53–57). For example, the disulfiram (DSF), a well-known antialcohol drug, combined with Cu triggered autophagic cell death and inhibited cell viability in colorectal cancer by targeting ULK1 (55). Tetrathiomolybdate (TM) and TPEN, specific Cu chelators, also showed obvious anti-tumor activity in COAD (58–60). In addition, quite a few novel Cu compounds were developed to investigate their antitumor mechanisms and therapeutic effect in COAD. For instance, the copper-imidazo[1,2-a] pyridines induced COAD apoptosis (61). Cu(dmp)_2_(CH_3_CN)]^2+^ exhibited anti-proliferative activity in human colorectal cancer cells (62). Cu(qmbn)(q)(Cl) triggered mitochondrion-mediated apoptotic cell death *via* activating the caspases-3 and 9 proteins (63). Moreover, nanoparticles combining Cu were designed to investigate the anticancer potential in COAD. Cu nanoparticles (CuNPs and Cu-Cy) shed a good insight for COAD treatment (64, 65). Cu_2_O@CaCO_3_ nanocomposites inhibited CRC distant metastasis and recurrence by immunotherapy through inducing an immunologically favorable TME and intensing the immune responses of anti-CD47 antibodies (66). The Bi : Cu_2_O@HA nanoparticles exhibited excellent targeting ability and photothermal therapeutic effect (67). Cuproptosis, a novel RCD, was recently identified as copper-dependent death, which occurred through directly binding Cu to TCA cycle (19). However, the role of cuproptosis in COAD is unclear, and the prognostic value of CRGs has not been thoroughly evaluated.

Thanks to the large public database such as TCGA and GEO, we are able to access and analyze the transcriptome profiles of a variety of malignancies to gain a comprehensive understanding of genetic landscape, screen potential biomarkers, develop therapy strategies and predict patient outcome (68, 69). Several studies have described cuproptosis-related molecular patterns and the characterization of TME in colorectal cancer and found that cuproptosis patterns were closely associated with TME and served to predicted survival of patients with colorectal cancer (70–74). D. Hou, et al. (75) developed a risk model of 11-cuproptosis-related lncRNAs to predict clinical and therapeutic implications of CRC patients. However, colon and rectal cancer were quite different in their biological characteristics, surgical protocol, treatment strategy and prognosis (76). Previously, Luo, B., et al. (77) identified two clusters based on 30 differentially expressed CRGs of 963 COAD samples from TCGA-COAD and GSE39582 databases. However, the OS between the two clusters showed no statistical difference and the accuracy of risk model was not verified. Xu, C., et al. (78) classified COAD samples from TCGA-COAD and GSE39582 databases into two groups according to 9 cuproptosis-related DEGs and further constructed a risk model. Whereas, ROC curves of the model showed that AUC values for the 1-year, 2-year, and 3-year survival were 0.575, 0.577 and 0.571 respectively, signifying the moderate predictive power of the model. In addition, Yang, G., et al. (79) grouped 623 COAD patients from TCGA-COAD and GSE17536 databases into 2 sets based on 12 CRGs expression profiles and established nomogram pattern based on risk model to predict patient prognosis. However, the sensitivity and specificity of the nomogram was not verified. As a result, we aimed to establish a more accurate risk model to predict survival through comprehensively integrating CRGs expression patterns of 1307 COAD samples from TCGA-COAD, GSE17536, GSE29623 and GSE39582 databases. In our study, 7 of 10 CRGs were found to be dys-regulated in tumor samples compared with those in normal samples, and a relatively high mutation frequency and CNV of CRGs was observed in COAD samples. Survival analysis and univariate Cox regression analysis of patients from TCGA (TCGA-COAD) and GEO database (GSE17536, GSE29623 and GSE39582) suggested both GLS and CDKN2A were significantly related with patients’ survival. The cuproptosis network demonstrated the complex interrelations among CRGs and prognosis of cancer patients. Considering the relatively common genetic and transcriptional variation and the potential prognostic value of CRGs in COAD, we speculated cuproptosis may be a new therapeutic target and that CRGs characteristics might play an important role in predicting therapeutic response and patient outcome, providing new insights into the role of Cu in COAD. We further categorized patients into three cuproptosis related molecular subtype, including subtype A, B and C, based on CRGs expression profile. Distinct molecular subtypes differed in both the CRGs expression profile, and the survival and clinicopathological features of COAD patients. GO and KEGG GSVA enrichment analyses suggested that different molecular subtypes enriched in different signaling pathways. Given the indispensable role of immunotherapy in colorectal adenocarcinoma, TIME-associated indicators such as TICs, MSI, CSC, TMB, somatic mutations, etc., were investigated to study the relationship between CRGs and TIME of colorectal adenocarcinoma. TICs profile revealed great difference in the infiltration of 19 TICs among distinct subtypes. GO enrichment analysis of 186 DEGs, obtained from the comparison between subtype A and B, A and C, and B and C, revealed that DEGs mainly enriched in signaling pathways associated with digestion. Univariate Cox regression analysis identified 86 prognostic DEGs from the above 186 genes. Based on 86 prognostic DEGs expression profile, we once again classified patients into 3 sets, namely gene subtype A, B and C, which were differed in the expressions of both prognostic DEGs and 8 CRGs. Additionally, cuproptosis gene subtypes were closely associated with the survival and clinicopathological characteristics (age, sex, grade, T and N stage) of COAD patients. In view of the important role of CRGs in COAD, the risk scoring system of CRG was further constructed in the training set according to prognostic DEGs expression, and verified in the testing set and the combined set. Risk scores of molecular subtype C and gene subtype C were significantly increased, while risk scores of molecular subtype B and gene subtype B were significantly decreased. The higher the risk score, the lower the survival rate. In addition, CRGs, TICs, CSC, TMB, MSI, somatic mutations, and drug sensitivity were closely associated with distinct risk score sets. Finally, a nomogram integrating risk scores and clinicopathological characteristics was established to predict OS rates of COAD patients. AUC values of 1-, 3-, and 5-year survival rates of nomogram were 0.873, 0.798, and 0.804, respectively, which was higher than previous nomogram established by Zhong, L., et al. (80). However, our study of the relationships between CRGs and TME in COAD were primarily based on the bioinformatics analysis. The specific mechanism of CRGs affecting TME needs to be further studied *in vitro* and *in vivo*, which may be crucial for the treatment of COAD.

## 5 Conclusion

CRGs were significantly correlated with clinicopathologic features, TME and immunoinfiltration of COAD. The higher the Risk score, the higher the MSI and TMB, and the lower the CSC. In addition, the CRGs Risk scoring system showed good ability to predict patient survival and drug sensitivity.

## Data availability statement

The original contributions presented in the study are included in the article/**Supplementary Material**. Further inquiries can be directed to the corresponding author.

## Ethics statement

The studies involving human participants were reviewed and approved by the Ethics Committee of Nanjing Jiangning Hospital. The patients/participants provided their written informed consent to participate in this study.

## Author contributions

(I) Conception and design: JW; ZT; (II) Administrative support: JW; ZT; BW; XH (III) Provision of study materials or patients: JW; XQ; DQ (IV) Collection and assembly of data: JW; YW; BL; DQ (V) Data analysis and interpretation: JW; YX; JC (VI) Manuscript writing: JW; (VII) All authors contributed to the article and approved the submitted version. 

## Supplementary material

Supplementary Figure 1 Unsupervised clustering of CRGs and consensus matrix heat-maps for k = 2, 4-9 through consensus clustering analysis in COAD samples from TCGA and GEO database.

Supplementary Figure 2 Unsupervised clustering of prognostic genes and consensus matrix heat-maps for k = 2, 4-9 through consensus clustering analysis in COAD samples from TCGA and GEO database.

Supplementary Figure 3 Identification of optimum prognostic genes in COAD samples. **(A, B)** The LASSO regression analysis and partial likelihood deviance analysis on 86 subtype-related prognostic DEGs.

Supplementary Figure 4 Difference, paired difference and survival analyses of 7 key Risk scoring genes (GLS, NOX1, HOXC6, TNNT1, PLA2G12B, CAB39L and ASRGL1) in COAD patients.

Supplementary Figure 5 Validation of CRG Risk score in the testing group. **(A)** Alluvial diagram of patients’ distributions in testing groups with different molecular subtypes, gene subtypes, Risk scores and survival outcomes. **(B)** Differential analysis of CRG Risk score in different molecular subtypes of the testing group. **(C)** Differential analysis of CRG Risk score in different gene subtypes of the testing group. **(D)** The heat-map of seven scoring genes expression in different risk sets of the testing group. **(E)** Ranked dot and scatter plot of CRG Risk score distribution and patient survival in the testing group. **(F)** Survival analysis of high- and low- CRG Risk score in the testing group. Kaplan–Meier plot and log-rank tests were conducted for survival analyses. P-value < 0.05 was considered to be statistically significant. **(G)** ROC curve predicted the sensitivity and specificity of 1-, 3-, and 5-year survival according to CRG Risk score in the testing group.

Supplementary Figure 6 Validation of CRG Risk score in GSE29623. **(A)** Alluvial diagram of patients’ distributions in testing groups with different molecular subtypes, gene subtypes, Risk scores and survival outcomes. **(B)** Differential analysis of CRG Risk score in different molecular subtypes of GSE29623. **(C)** Differential analysis of CRG Risk score in different gene subtypes of GSE29623. **(D)** The heat-map of seven scoring genes expression in different risk sets of GSE29623. **(E)** Ranked dot and scatter plot of CRG Risk score distribution and patient survival in GSE29623. **(F)** Survival analysis of high- and low- CRG Risk score in GSE29623. Kaplan–Meier plot and log-rank tests were conducted for survival analyses. P-value< 0.05 was considered to be statistically significant. **(G)** ROC curve predicted the sensitivity and specificity of 1-, 3-, and 5-year survival according to CRG Risk score in GSE29623.

Supplementary Figure 7 Validation of CRG Risk score in GSE17536. **(A)** Alluvial diagram of patients’ distributions in testing groups with different molecular subtypes, gene subtypes, Risk scores and survival outcomes. **(B)** Differential analysis of CRG Risk score in different molecular subtypes of GSE17536. **(C)** Differential analysis of CRG Risk score in different gene subtypes of GSE17536. **(D)** The heat-map of seven scoring genes expression in different risk sets of GSE17536. **(E)** Ranked dot and scatter plot of CRG Risk score distribution and patient survival in GSE17536. **(F)** Survival analysis of high- and low- CRG Risk score in GSE17536. Kaplan–Meier plot and log-rank tests were conducted for survival analyses. P-value< 0.05 was considered to be statistically significant. **(G)** ROC curve predicted the sensitivity and specificity of 1-, 3-, and 5-year survival according to CRG Risk score in GSE17536.

Supplementary Figure 8 Validation of CRG Risk score in GSE39582. **(A)** Alluvial diagram of patients’ distributions in testing groups with different molecular subtypes, gene subtypes, Risk scores and survival outcomes. **(B)** Differential analysis of CRG Risk score in different molecular subtypes of GSE39582. **(C)** Differential analysis of CRG Risk score in different gene subtypes of GSE39582. **(D)** The heat-map of seven scoring genes expression in different risk sets of GSE39582. **(E)** Ranked dot and scatter plot of CRG Risk score distribution and patient survival in GSE39582. **(F)** Survival analysis of high- and low- CRG Risk score in GSE39582. Kaplan–Meier plot and log-rank tests were conducted for survival analyses. P-value< 0.05 was considered to be statistically significant. **(G)** ROC curve predicted the sensitivity and specificity of 1-, 3-, and 5-year survival according to CRG Risk score in GSE39582.

Supplementary Figure 9 Validation of CRG Risk score in GSE40967. **(A)** The heat-map of seven scoring genes expression in different risk sets of the combined group. **(B)** Ranked dot and scatter plot of CRG Risk score distribution and patient survival in the combined group. **(C)** Survival analysis of high- and low- CRG Risk score in the combined group. Kaplan–Meier plot and log-rank tests were conducted for survival analyses. P-value< 0.05 was considered to be statistically significant. **(D)** ROC curve predicted the sensitivity and specificity of 1-, 3-, and 5-year survival according to CRG Risk score in the combined group.

Supplementary Figure 10 Differential drugs susceptibility analyses in high- and low-Risk group. P-value< 0.05 was considered to be statistically significant.

Supplementary Figure 11 Differential drugs susceptibility analyses in high- and low-Risk group. P-value< 0.05 was considered to be statistically significant.
