# Supplementary figures and images for: Cuproptosis-related risk score predicts prognosis and characterizes the tumor microenvironment in colon adenocarcinoma
Source: Front Oncol. 2023 Jun 2;13:1152681. doi: 10.3389/fonc.2023.1152681 (PMC10272849; doi:10.3389/fonc.2023.1152681)

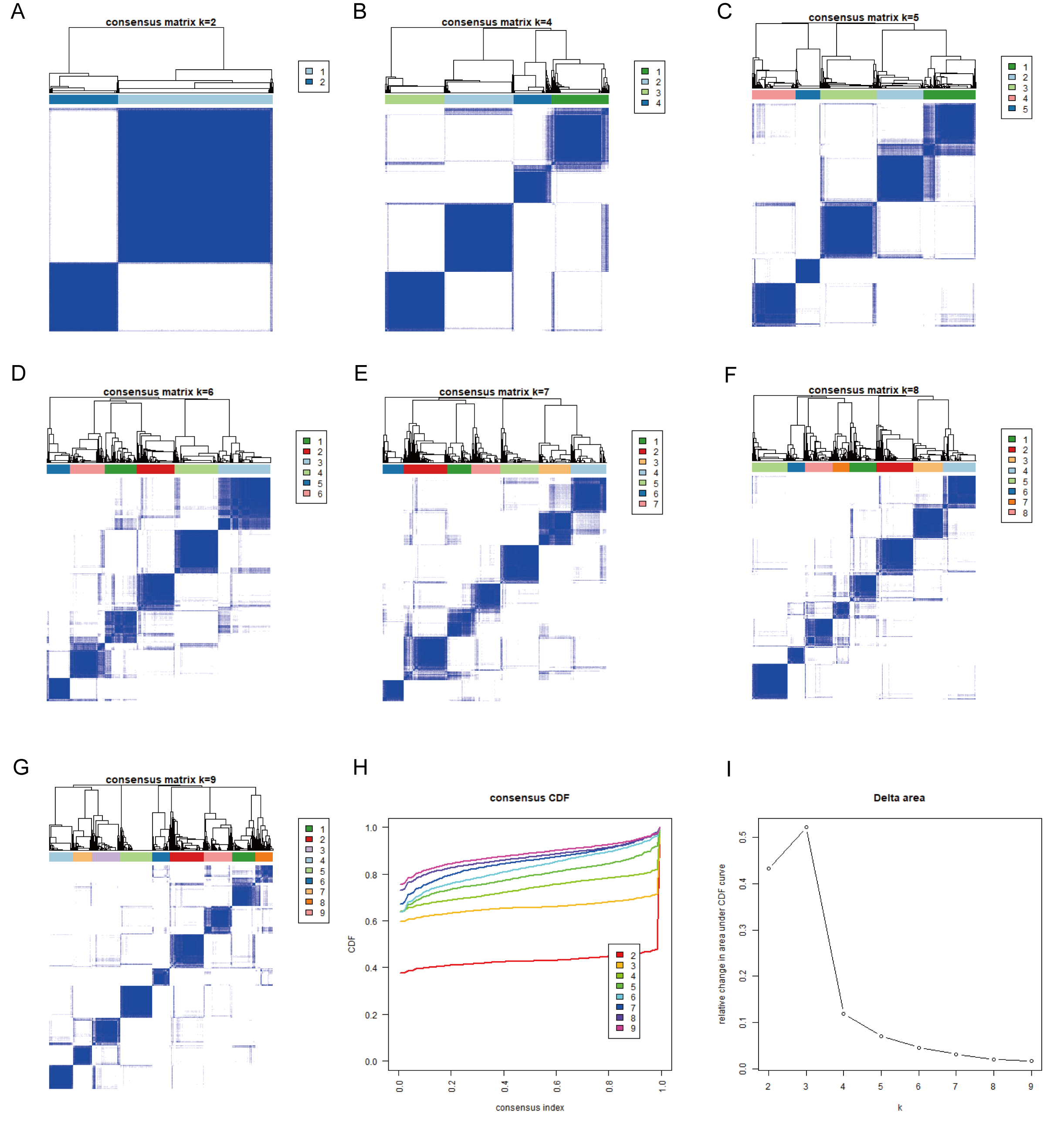

Supplement: Supplementary Figure 1 — Unsupervised clustering of CRGs and consensus matrix heat-maps for k = 2, 4-9 through consensus clustering analysis in COAD samples from TCGA and GEO database. [file Image_1.tif]

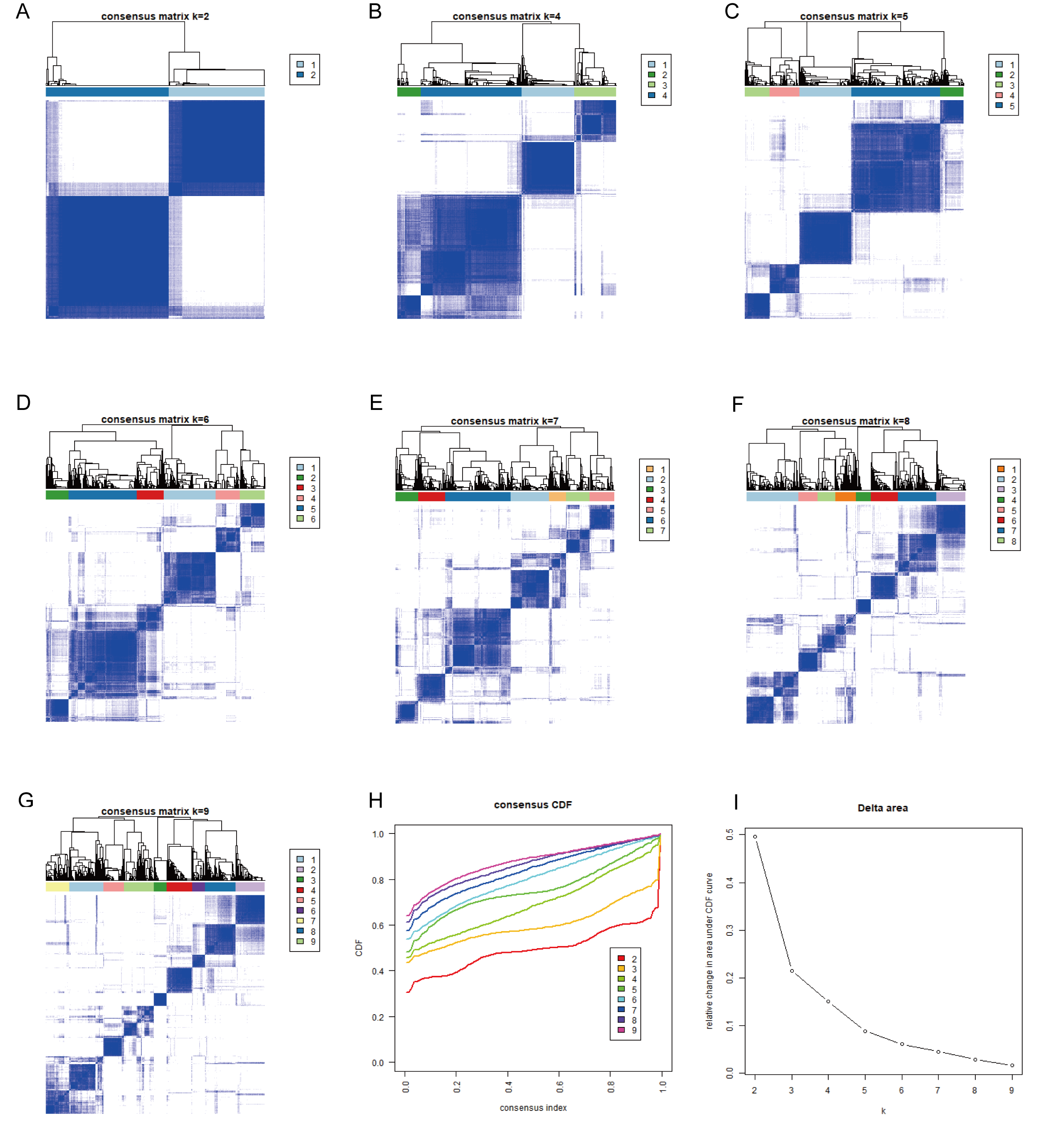

Supplement: Supplementary Figure 2 — Unsupervised clustering of prognostic genes and consensus matrix heat-maps for k = 2, 4-9 through consensus clustering analysis in COAD samples from TCGA and GEO database. [file Image_2.tif]

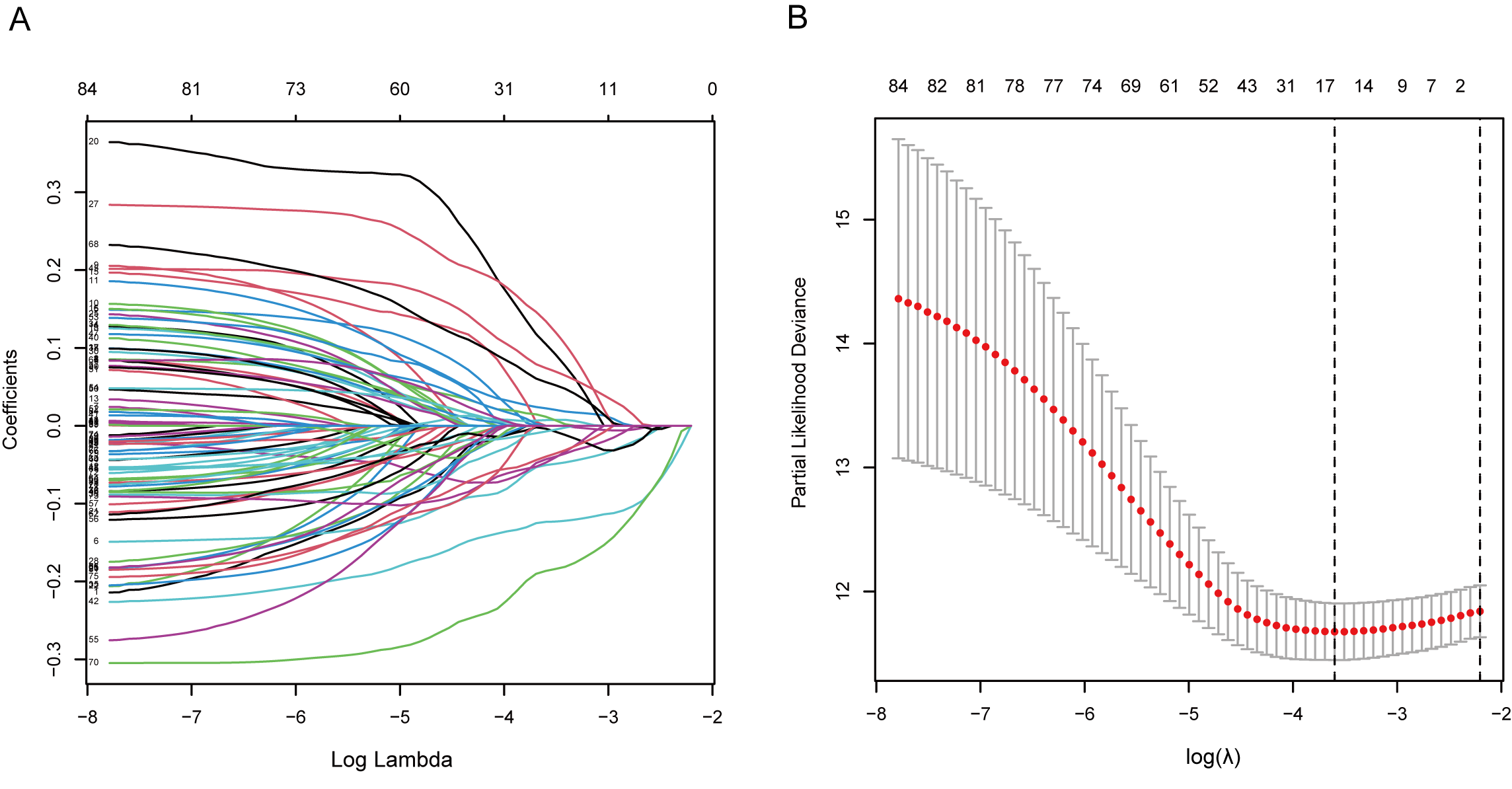

Supplement: Supplementary Figure 3 — Identification of optimum prognostic genes in COAD samples. (A, B) The LASSO regression analysis and partial likelihood deviance analysis on 86 subtype-related prognostic DEGs. [file Image_3.tif]

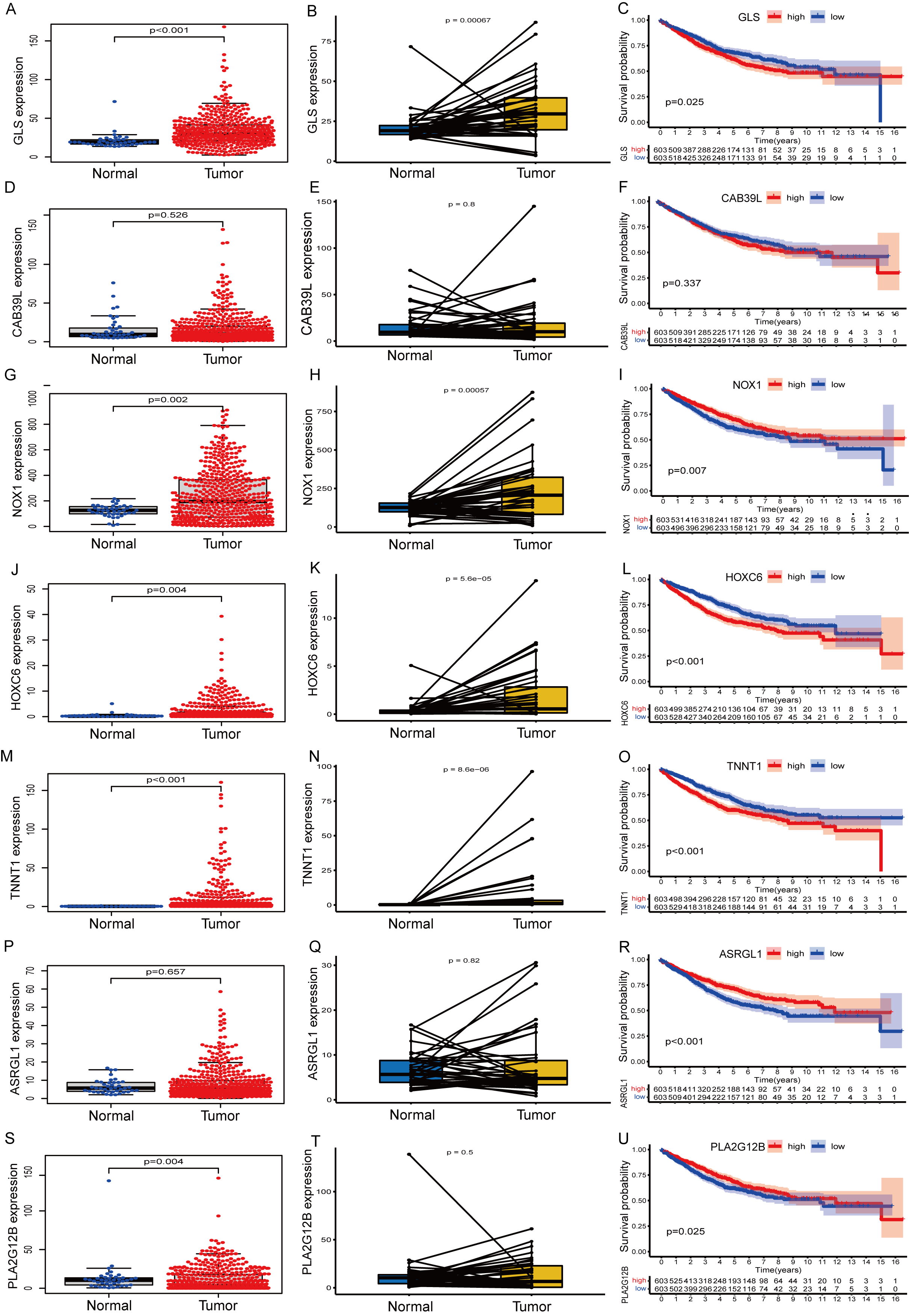

Supplement: Supplementary Figure 4 — Difference, paired difference and survival analyses of 7 key Risk scoring genes (GLS, NOX1, HOXC6, TNNT1, PLA2G12B, CAB39L and ASRGL1) in COAD patients. [file Image_4.tif]

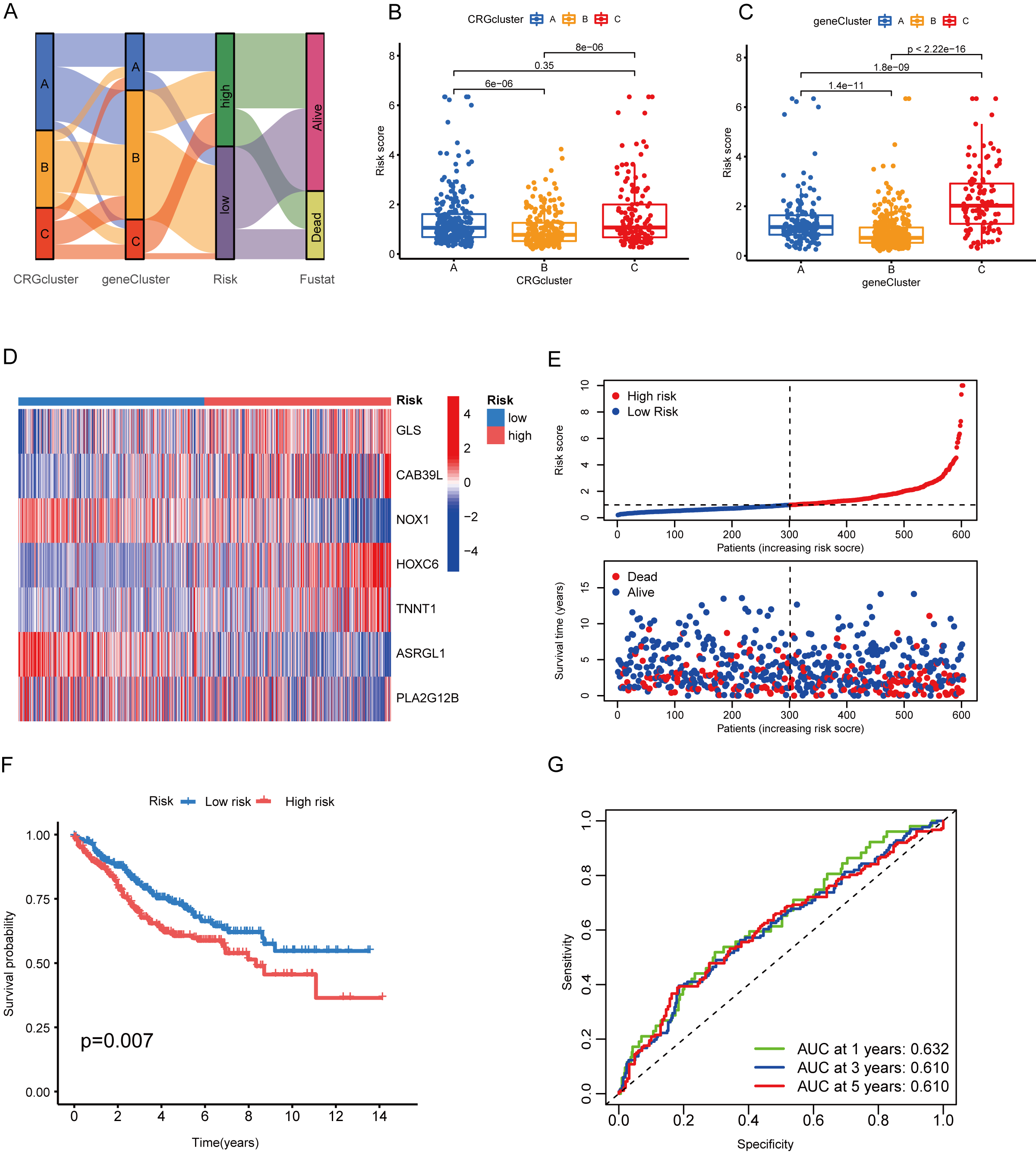

Supplement: Supplementary Figure 5 — Validation of CRG Risk score in the testing group. (A) Alluvial diagram of patients’ distributions in testing groups with different molecular subtypes, gene subtypes, Risk scores and survival outcomes. (B) Differential analysis of CRG Risk score in different molecular subtypes of the testing group. (C) Differential analysis of CRG Risk score in different gene subtypes of the testing group. (D) The heat-map of seven scoring genes expression in different risk sets of the testing group. (E) Ranked dot and scatter plot of CRG Risk score distribution and patient survival in the testing group. (F) Survival analysis of high- and low- CRG Risk score in the testing group. Kaplan–Meier plot and log-rank tests were conducted for survival analyses. P-value < 0.05 was considered to be statistically significant. (G) ROC curve predicted the sensitivity and specificity of 1-, 3-, and 5-year survival according to CRG Risk score in the testing group. [file Image_5.tif]

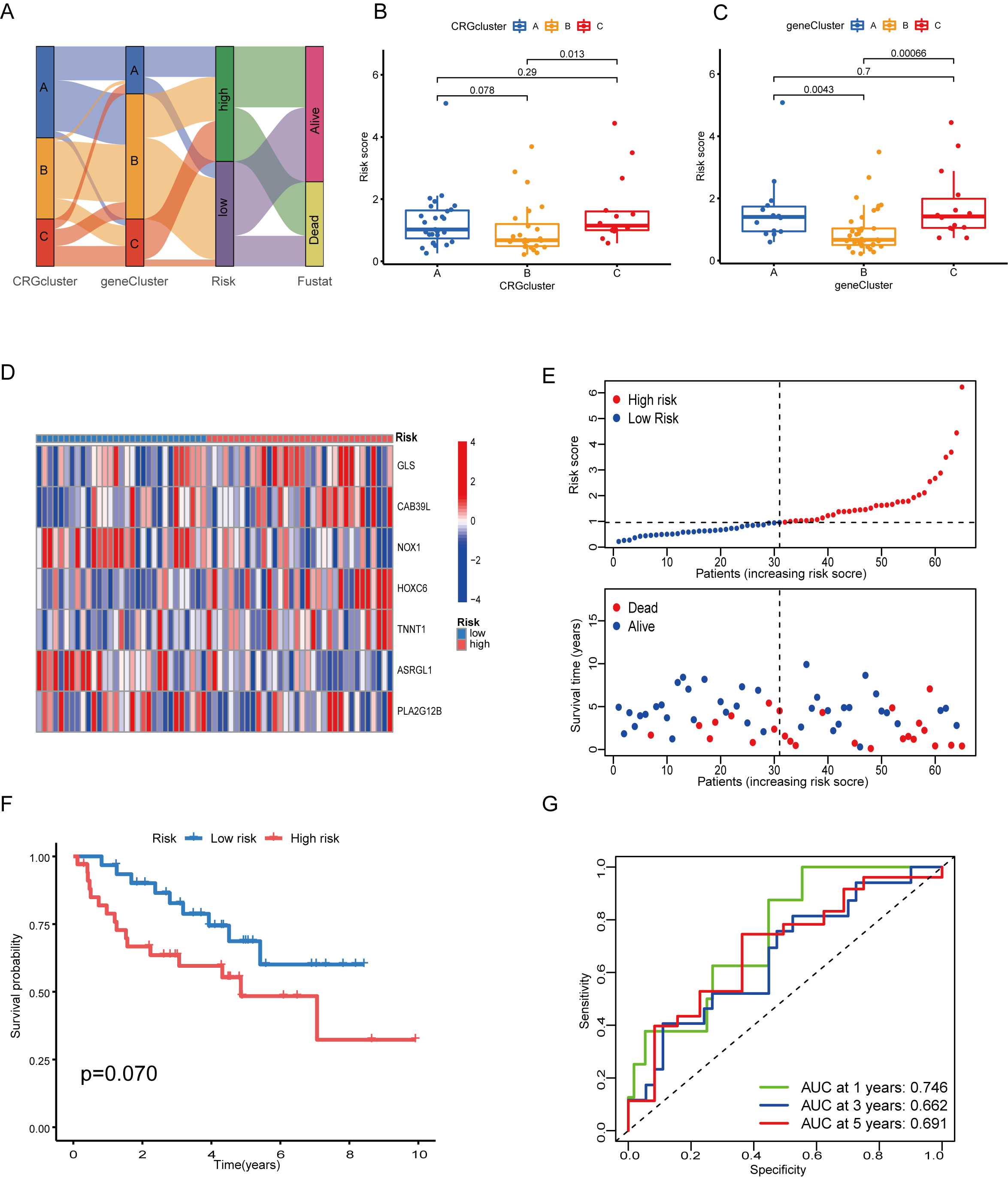

Supplement: Supplementary Figure 6 — Validation of CRG Risk score in GSE29623. (A) Alluvial diagram of patients’ distributions in testing groups with different molecular subtypes, gene subtypes, Risk scores and survival outcomes. (B) Differential analysis of CRG Risk score in different molecular subtypes of GSE29623. (C) Differential analysis of CRG Risk score in different gene subtypes of GSE29623. (D) The heat-map of seven scoring genes expression in different risk sets of GSE29623. (E) Ranked dot and scatter plot of CRG Risk score distribution and patient survival in GSE29623. (F) Survival analysis of high- and low- CRG Risk score in GSE29623. Kaplan–Meier plot and log-rank tests were conducted for survival analyses. P-value< 0.05 was considered to be statistically significant. (G) ROC curve predicted the sensitivity and specificity of 1-, 3-, and 5-year survival according to CRG Risk score in GSE29623. [file Image_6.tif]

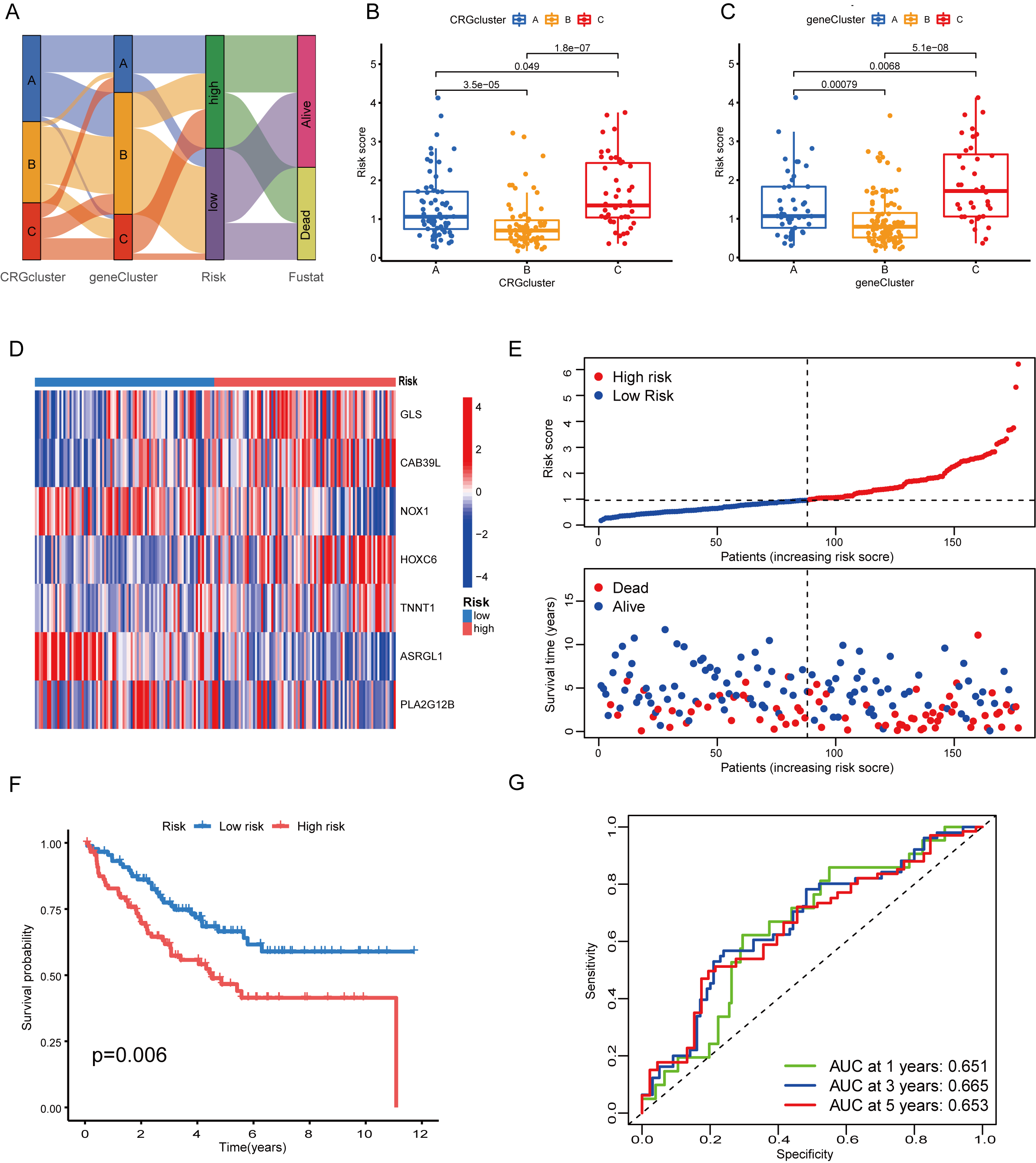

Supplement: Supplementary Figure 7 — Validation of CRG Risk score in GSE17536. (A) Alluvial diagram of patients’ distributions in testing groups with different molecular subtypes, gene subtypes, Risk scores and survival outcomes. (B) Differential analysis of CRG Risk score in different molecular subtypes of GSE17536. (C) Differential analysis of CRG Risk score in different gene subtypes of GSE17536. (D) The heat-map of seven scoring genes expression in different risk sets of GSE17536. (E) Ranked dot and scatter plot of CRG Risk score distribution and patient survival in GSE17536. (F) Survival analysis of high- and low- CRG Risk score in GSE17536. Kaplan–Meier plot and log-rank tests were conducted for survival analyses. P-value< 0.05 was considered to be statistically significant. (G) ROC curve predicted the sensitivity and specificity of 1-, 3-, and 5-year survival according to CRG Risk score in GSE17536. [file Image_7.tif]

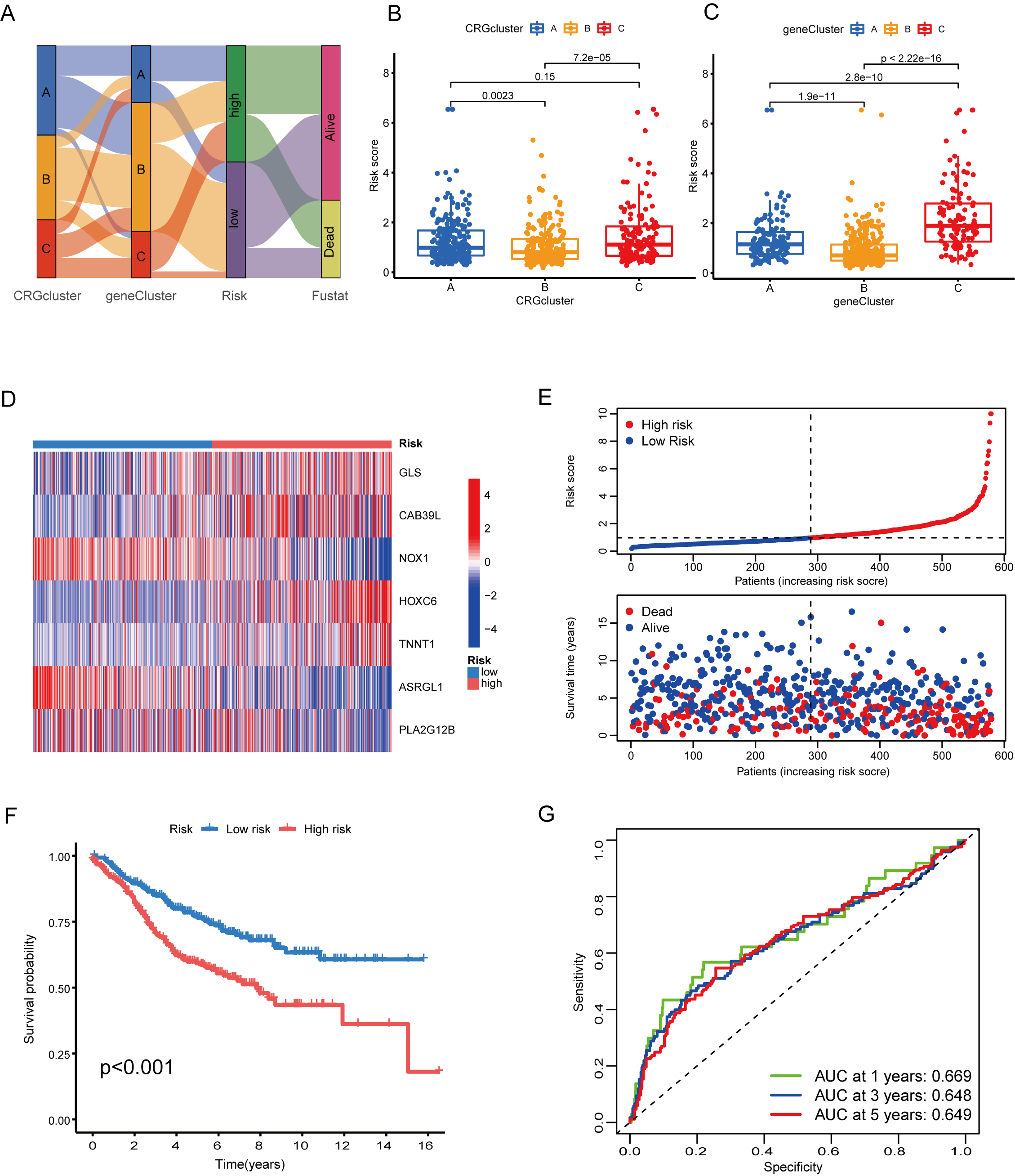

Supplement: Supplementary Figure 8 — Validation of CRG Risk score in GSE39582. (A) Alluvial diagram of patients’ distributions in testing groups with different molecular subtypes, gene subtypes, Risk scores and survival outcomes. (B) Differential analysis of CRG Risk score in different molecular subtypes of GSE39582. (C) Differential analysis of CRG Risk score in different gene subtypes of GSE39582. (D) The heat-map of seven scoring genes expression in different risk sets of GSE39582. (E) Ranked dot and scatter plot of CRG Risk score distribution and patient survival in GSE39582. (F) Survival analysis of high- and low- CRG Risk score in GSE39582. Kaplan–Meier plot and log-rank tests were conducted for survival analyses. P-value< 0.05 was considered to be statistically significant. (G) ROC curve predicted the sensitivity and specificity of 1-, 3-, and 5-year survival according to CRG Risk score in GSE39582. [file Image_8.tif]

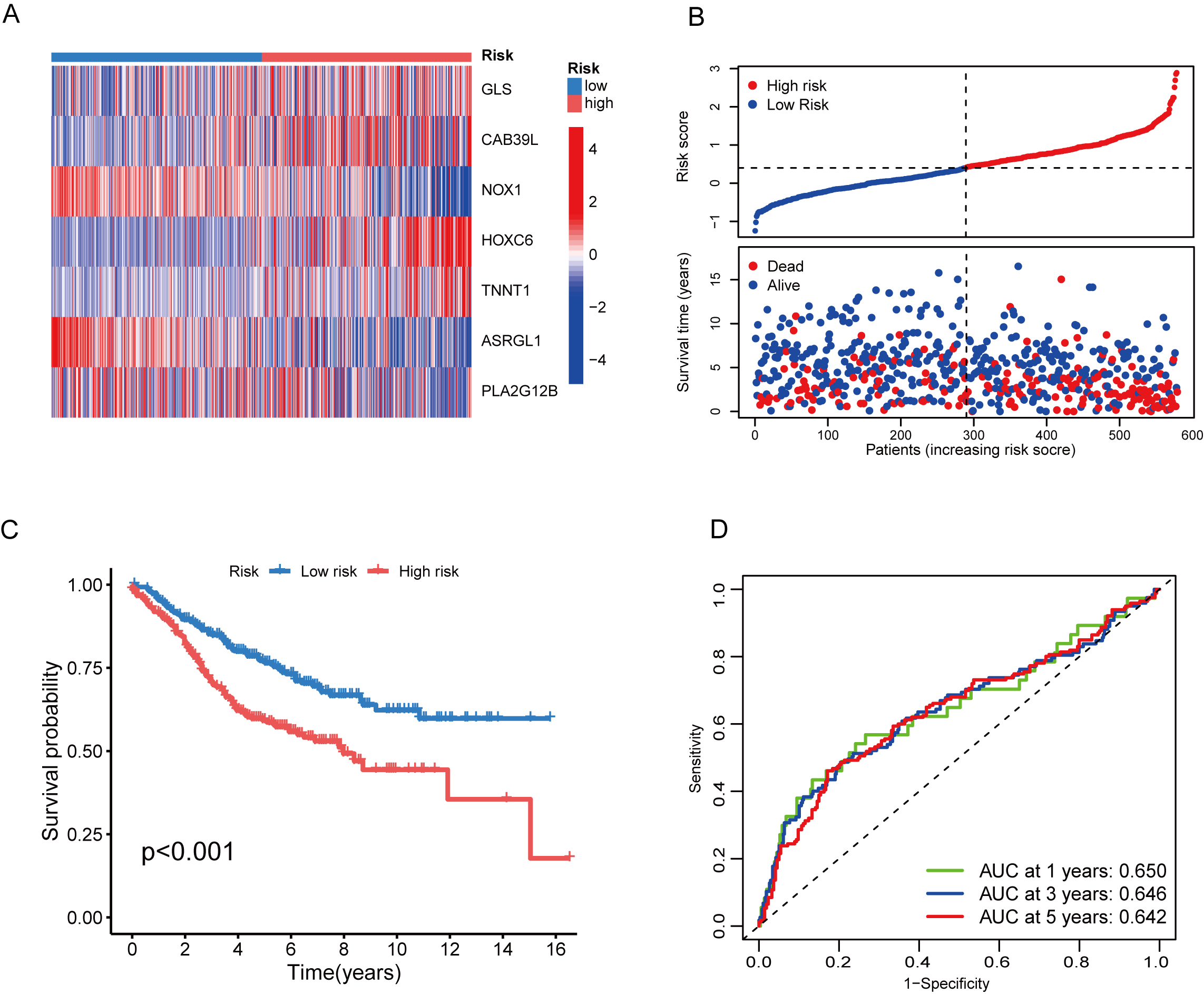

Supplement: Supplementary Figure 9 — Validation of CRG Risk score in GSE40967. (A) The heat-map of seven scoring genes expression in different risk sets of the combined group. (B) Ranked dot and scatter plot of CRG Risk score distribution and patient survival in the combined group. (C) Survival analysis of high- and low- CRG Risk score in the combined group. Kaplan–Meier plot and log-rank tests were conducted for survival analyses. P-value< 0.05 was considered to be statistically significant. (D) ROC curve predicted the sensitivity and specificity of 1-, 3-, and 5-year survival according to CRG Risk score in the combined group. [file Image_9.tif]

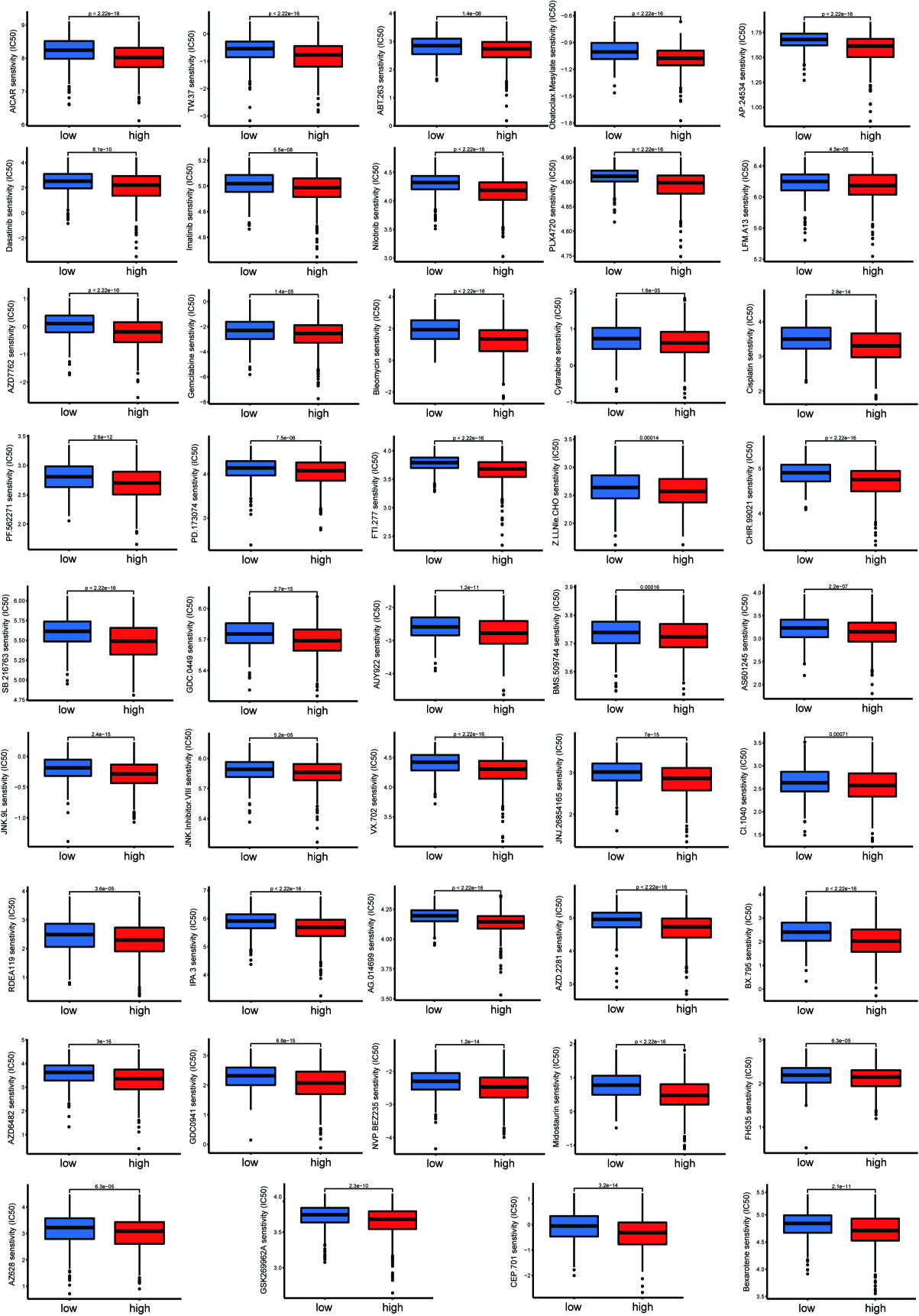

Supplement: Supplementary Figure 10 — Differential drugs susceptibility analyses in high- and low-Risk group. P-value< 0.05 was considered to be statistically significant. [file Image_10.tif]

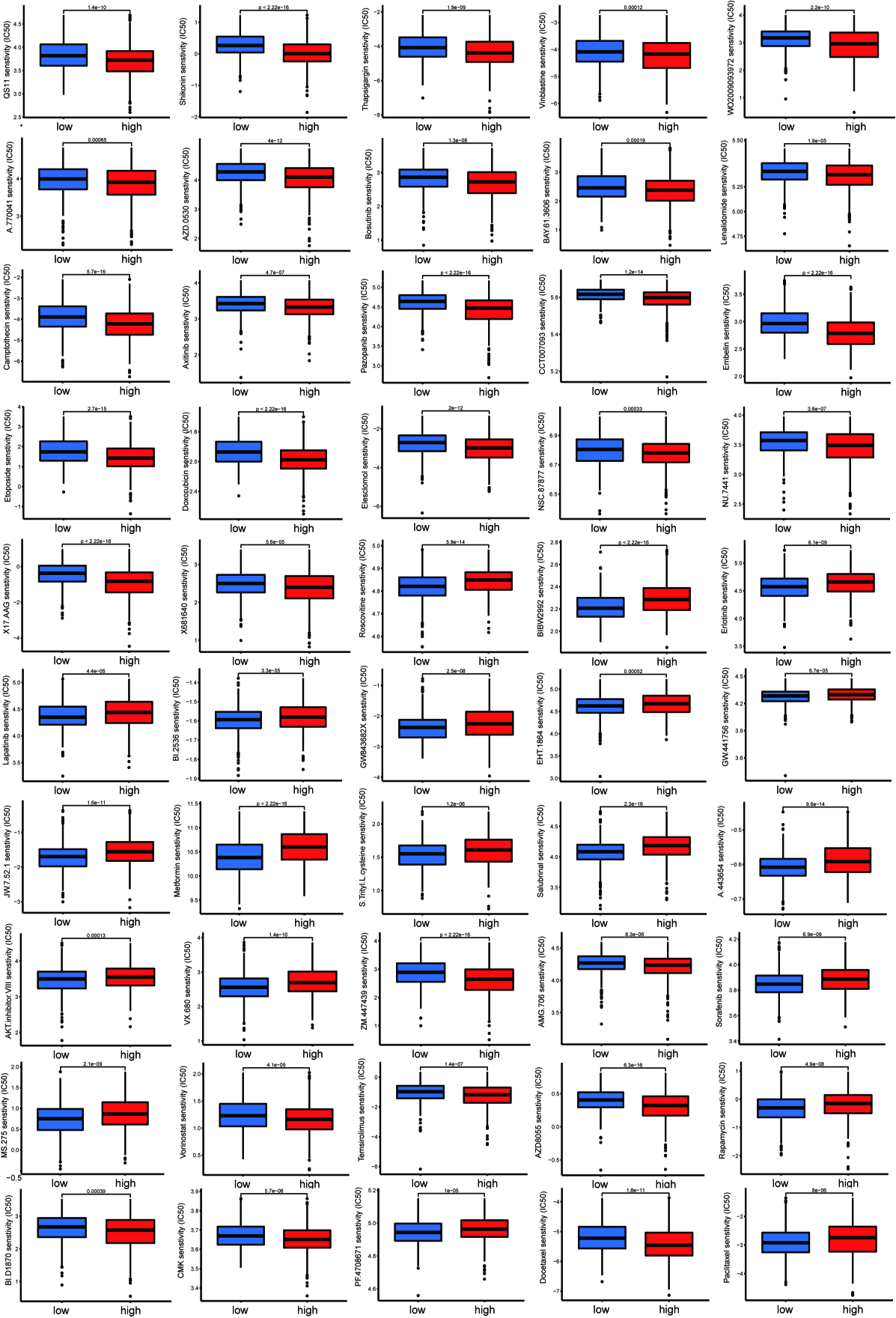

Supplement: Supplementary Figure 11 — Differential drugs susceptibility analyses in high- and low-Risk group. P-value< 0.05 was considered to be statistically significant. [file Image_11.tif]
